# The arginine methyltransferase Prmt1 coordinates the germline arginine methylome essential for spermatogonial homeostasis and male fertility

**DOI:** 10.1093/nar/gkad769

**Published:** 2023-09-22

**Authors:** Muhammad Azhar, Caoling Xu, Xue Jiang, Wenqing Li, Yuzhu Cao, Xiaoli Zhu, Xuemei Xing, Limin Wu, Jiaqi Zou, Lan Meng, Yu Cheng, Wenjie Han, Jianqiang Bao

**Affiliations:** Department of Obstetrics and Gynecology, Reproductive and Genetic Hospital, The First Affiliated Hospital of USTC, Division of Life Sciences and Medicine, University of Science and Technology of China, Hefei, Anhui 230001, China; Hefei National Laboratory for Physical Sciences at Microscale, Biomedical Sciences and Health Laboratory of Anhui Province, University of Science and Technology of China (USTC), Anhui, China; Department of Obstetrics and Gynecology, Reproductive and Genetic Hospital, The First Affiliated Hospital of USTC, Division of Life Sciences and Medicine, University of Science and Technology of China, Hefei, Anhui 230001, China; Hefei National Laboratory for Physical Sciences at Microscale, Biomedical Sciences and Health Laboratory of Anhui Province, University of Science and Technology of China (USTC), Anhui, China; Department of Obstetrics and Gynecology, Reproductive and Genetic Hospital, The First Affiliated Hospital of USTC, Division of Life Sciences and Medicine, University of Science and Technology of China, Hefei, Anhui 230001, China; Hefei National Laboratory for Physical Sciences at Microscale, Biomedical Sciences and Health Laboratory of Anhui Province, University of Science and Technology of China (USTC), Anhui, China; Department of Obstetrics and Gynecology, Reproductive and Genetic Hospital, The First Affiliated Hospital of USTC, Division of Life Sciences and Medicine, University of Science and Technology of China, Hefei, Anhui 230001, China; Hefei National Laboratory for Physical Sciences at Microscale, Biomedical Sciences and Health Laboratory of Anhui Province, University of Science and Technology of China (USTC), Anhui, China; Department of Obstetrics and Gynecology, Reproductive and Genetic Hospital, The First Affiliated Hospital of USTC, Division of Life Sciences and Medicine, University of Science and Technology of China, Hefei, Anhui 230001, China; Hefei National Laboratory for Physical Sciences at Microscale, Biomedical Sciences and Health Laboratory of Anhui Province, University of Science and Technology of China (USTC), Anhui, China; Department of Obstetrics and Gynecology, Reproductive and Genetic Hospital, The First Affiliated Hospital of USTC, Division of Life Sciences and Medicine, University of Science and Technology of China, Hefei, Anhui 230001, China; Hefei National Laboratory for Physical Sciences at Microscale, Biomedical Sciences and Health Laboratory of Anhui Province, University of Science and Technology of China (USTC), Anhui, China; Department of Obstetrics and Gynecology, Reproductive and Genetic Hospital, The First Affiliated Hospital of USTC, Division of Life Sciences and Medicine, University of Science and Technology of China, Hefei, Anhui 230001, China; Department of Obstetrics and Gynecology, Reproductive and Genetic Hospital, The First Affiliated Hospital of USTC, Division of Life Sciences and Medicine, University of Science and Technology of China, Hefei, Anhui 230001, China; Department of Obstetrics and Gynecology, Reproductive and Genetic Hospital, The First Affiliated Hospital of USTC, Division of Life Sciences and Medicine, University of Science and Technology of China, Hefei, Anhui 230001, China; Hefei National Laboratory for Physical Sciences at Microscale, Biomedical Sciences and Health Laboratory of Anhui Province, University of Science and Technology of China (USTC), Anhui, China; Department of Obstetrics and Gynecology, Reproductive and Genetic Hospital, The First Affiliated Hospital of USTC, Division of Life Sciences and Medicine, University of Science and Technology of China, Hefei, Anhui 230001, China; Hefei National Laboratory for Physical Sciences at Microscale, Biomedical Sciences and Health Laboratory of Anhui Province, University of Science and Technology of China (USTC), Anhui, China; Department of Obstetrics and Gynecology, Reproductive and Genetic Hospital, The First Affiliated Hospital of USTC, Division of Life Sciences and Medicine, University of Science and Technology of China, Hefei, Anhui 230001, China; Hefei National Laboratory for Physical Sciences at Microscale, Biomedical Sciences and Health Laboratory of Anhui Province, University of Science and Technology of China (USTC), Anhui, China; Hefei National Laboratory for Physical Sciences at Microscale, Biomedical Sciences and Health Laboratory of Anhui Province, University of Science and Technology of China (USTC), Anhui, China; Department of Obstetrics and Gynecology, Reproductive and Genetic Hospital, The First Affiliated Hospital of USTC, Division of Life Sciences and Medicine, University of Science and Technology of China, Hefei, Anhui 230001, China; Hefei National Laboratory for Physical Sciences at Microscale, Biomedical Sciences and Health Laboratory of Anhui Province, University of Science and Technology of China (USTC), Anhui, China

## Abstract

Arginine methylation, catalyzed by the protein arginine methyltransferases (PRMTs), is a common post-translational protein modification (PTM) that is engaged in a plethora of biological events. However, little is known about how the methylarginine-directed signaling functions in germline development. In this study, we discover that Prmt1 is predominantly distributed in the nuclei of spermatogonia but weakly in the spermatocytes throughout mouse spermatogenesis. By exploiting a combination of three Cre-mediated Prmt1 knockout mouse lines, we unravel that Prmt1 is essential for spermatogonial establishment and maintenance, and that Prmt1-catalyzed asymmetric methylarginine coordinates inherent transcriptional homeostasis within spermatogonial cells. In conjunction with high-throughput CUT&Tag profiling and modified mini-bulk Smart-seq2 analyses, we unveil that the Prmt1-deposited H4R3me2a mark is permissively enriched at promoter and exon/intron regions, and sculpts a distinctive transcriptomic landscape as well as the alternative splicing pattern, in the mouse spermatogonia. Collectively, our study provides the genetic and mechanistic evidence that connects the Prmt1-deposited methylarginine signaling to the establishment and maintenance of a high-fidelity transcriptomic identity in orchestrating spermatogonial development in the mammalian germline.

## Graphical Abstract

**Graphical Abstract:**
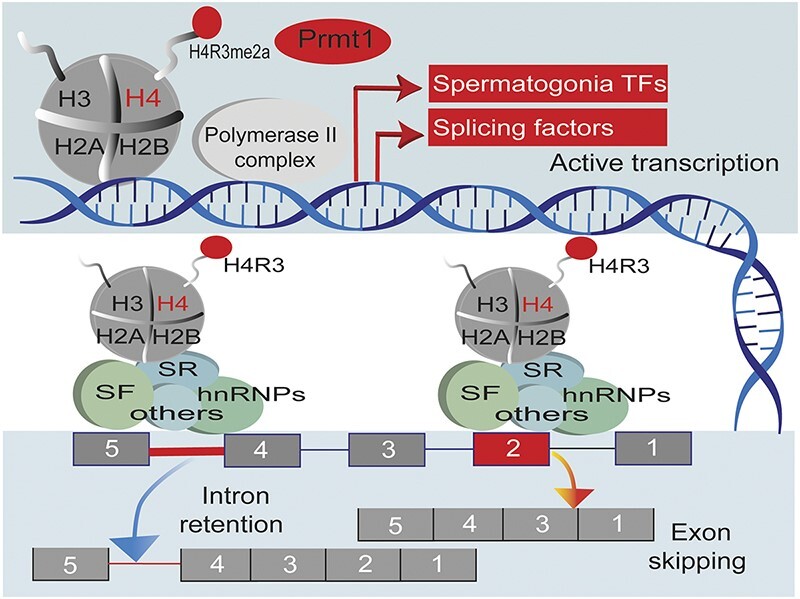

## INTRODUCTION

In mammals, spermatogenesis is a spatiotemporally organized and complex developmental process that takes place in the epithelium of seminiferous tubules in the testis. In general, this process can be divided into three successive processes, including (i) mitotic proliferation of spermatogonial stem cells (SSCs) and progenitor spermatogonia, and subsequent differentiation giving rise to differentiated spermatogonia (1); (ii) meiotic division with one-time duplication of chromosomes followed by two times of nuclear divisions (2) and (iii) spermiogenesis, in which the haploid round spermatids undergo morphological transformation and nuclear condensation (3). The production of competent sperm necessitates rigorous gene expression regulation at both the transcriptional and post-transcriptional levels. A wide range of post-transcriptional RNA modifications and histone post-translational modifications (PTMs) was present in spermatogonia, spermatocytes and spermatids in a stage-specifically regulated manner. For instance, the most abundant N^6^-methyladenosine (m^6^A) modification in mRNA deposited by the Mettl3/14 complex is functionally important for the translation of m^6^A-carrying mRNAs required for SSC proliferation and spermatogonial differentiation. Integrative multiparametric analyses revealed that a variety of histone lysine modification marks (e.g. histone H3 lysine 4 methylation, lysine 27 methylation and acetylation) occur in spermatogonia that epigenetically distinguish their sub-stages throughout spermatogonial development in developing mouse testes (4–6).

Arginine methylation is such one common PTM that is abundantly present in both histone and non-histone proteins. The initial high-performance liquid chromatography (HPLC)-based quantification estimated that ∼0.5–2% of total arginine residues are methylated in mammalian cells and tissues. A recent global profiling by label-free NMR spectroscopy, independent of chromatography separation, has identified the methylated arginine proportion ranging from 1% to 3.4% in human cell lines (7). Arginine methylation is catalyzed by the family of protein arginine methyltransferases (PRMTs), which deposit three types (I∼III) of methyl marks on arginine residues, including ω-N^G^, N^G^ asymmetric dimethylarginine (ADMA), ω-N^G^, N^G^-symmetric dimethylarginine (SDMA) and ω-N^G^-monomethylarginine (MMA), and accordingly, they belong to the Type I (PRMT1/2/3/4/6/8), Type II (PRMT5/9) and Type III (PRMT7) enzymes, respectively (8). Of note, all three types of PRMTs could deposit the MMA mark on substrates and the MMA modification is generally regarded as an intermediate state prior to further addition of a methyl mark by Type I or Type II PRMT enzymes, which thereby converts MMA to ADMA and SDMA, respectively (9). Most PRMTs prefer to methylate glycine- and arginine-rich, consensus peptides, known as so-called GAR (RG/RGG/RXG) motifs (10). These motifs were found in both histone and non-histone protein substrates that support both ADMA and SDMA marks. Noteworthily, PRMT4, also known as CARM1, displays unique substrate specificity within proline-, glycine-, and methionine-rich regions (termed the PGM motif) (11). On the other hand, PRMT5 can symmetrically dimethylate arginine residues within either GAR or PGM motifs (9), suggesting an intricate cross-talk of substrate methylation among three different types of PRMTs. However, owing to the presence of multiple neighboring arginine residues within the GAR or PGM motifs, it is currently unclear to what extent for the different types of methyl marks (MMA, ADMA and SDMA) to functionally compete with each other.

Mechanistically, the methyl marks serve as ‘docking sites’ for recognition by ‘reader’ proteins, which specifically recognize this mark and, upon recognition/interaction, activate the downstream signaling directly or indirectly through the recruitment of protein readers/partners. Except for PRMT8, which is restricted to neuronal cells (12), most PRMTs are ubiquitously expressed in mammalian tissues. To date, arginine methylation has been widely implicated in a broad range of biological processes involving cell growth, proliferation, and differentiation, and the deficient methylarginine signaling is closely linked to cellular transformation or developmental deficiency. For instance, we have shown that Carm1 is highly enriched in the nuclei of haploid spermatids in the mouse testis, and its loss leads to severe defects in the elongation and condensation of spermatids during spermiogenesis, resulting in male infertility (13). Prmt5 is abundantly expressed in the embryonic and postnatal germline, and deposits the symmetrical dimethylation of H4R3 (H4R3me2s), which is critical for the development of primordial germ cells (PGCs) and postnatal meiotic divisions of spermatocytes by maintaining the genome integrity likely through the Piwi–piRNA pathway in the mouse testis (14). Prmt7 is a unique member of the PRMT family in that it is solely responsible for the arginine mono-methylation (15). While it is not required for postnatal germ cell development, it is crucial to promote germ cell proliferation in the embryonic testis through the BMP and TGF-β signaling pathway, and is implicated in paternal genomic imprinting. Prmt1 (16) and Prmt6 (17) have previously been implicated in spermatogenesis and male fertility. The mice with global loss of Prmt3 (18) or Prmt6 (19) showed normal gross morphology, implying that Prmt3/6 are compatible with germ cell survival/development, or there might be redundant roles from other PRMT members.

In mammalian cells, Prmt1 is the primary Type I methyltransferase responsible for ADMA deposition. Herein, we showed that the Prmt1 protein is abundantly expressed in postnatal mouse testicular germ cells throughout spermatogenesis, with the highest levels enriched in the nuclei of spermatogonia. The germline-specific Stra8-cre mediated inactivation of Prmt1 caused only the death of meiotic spermatocytes without any detectable defects in spermatogonia owing to the inefficiency of two Loxp-cassette allele excisions by germline Cre. By using two tamoxifen-inducible Cre lines [Ubc-CreERT2*; Prmt1^loxp/lox^* (Prmt1-uKO), and Ddx4-CreERT2; *Prmt1^loxp/lox^* (Prmt1-dKO)], we unexpectedly found that the substrate methylation levels for ADMA, SDMA and MMA were together markedly elevated in germ cells, which is different from those observed in somatic cells. Transcriptome-wide and CUT&Tag epigenomic profiling for five histone methylarginine marks uncovered that the pre-defined H4R3me2a by Prmt1 predominantly occupies both the gene promoter and intronic/exonic regions, which orchestrates the expression of a large number of splicing and splicing-related factors as well as maintains local chromatin signature, in spermatogonial cells. Together, our study reveals an essential role of methylarginine in coordinating spermatogonial development, and provides mechanistic evidence that links the methylarginine signaling to shaping the spermatogonial transcriptome indispensable for male fertility.

## MATERIALS AND METHODS

### Animal use and care

All of the mice utilized in this study were in the C57BL/6J genetic background. To study the effect of *Prmt1* abrogation on germ cells, we generated floxed *Prmt1* (*Prmt1*^lox/lox^) alleles (GemPharmatech Co., Ltd). Based on the Ensemble database, the *Prmt1* locus encodes 14 transcripts in total. Exons 4 and 5 are conserved across all transcripts and were thus chosen for Loxp cassette targeting through the CRISPR/Cas9 strategy. Briefly, a pair of sgRNAs were transcribed *in vitro* and subsequently injected into mouse zygotes along with a donor vector to generate F0 chimera offspring. The loxp-positive line was confirmed via PCR genotyping and crossed with C57BL/6J mice to attain the F1 generation of *Prmt1*^lox^ allele. The Stra8-GFPCre knock-in (KI) mouse strain (gift from Ming-Han Tong's lab at the Chinese Academy of Sciences, Shanghai), the Zp3-Cre line (purchased from Jackson Laboratory) and the Ubc-CreERT2 KI mice (purchased from GemPharmatech Co., Ltd) were crossed with *Prmt1*^lox/lox^ to attain Prmt1-sKO, Prmt1-zKO and Prmt1-uKO progenies, respectively. To bypass the embryonic lethality and to manipulate the gene deletion in a stage-dependent manner, the Ddx4-CreERT2 KI mouse strain (*C57BL/6J-Ddx4^tm1(5×HA-P2A-EGFP-T2A-CreERT2)Bao^*), wherein the Ddx4 promoter drives the expression of C-terminally 5 × HA-tagged Ddx4 fusion protein, along with EGFP and CreERT2 fusion enzyme in the germline, simultaneously, was generated and characterized *in-house* (manuscript in preparation). Upon the manual injection of tamoxifen (Tam), the released CreERT2 fusion protein enters the nucleus and excises the gene fragment flanked by a pair of Loxp cassettes, leading to the production of inducible Prmt1 KO (Prmt1-dKO). All mice were bred in a 12-h light/dark cycle and with free access to food and water in a specific pathogen-free facility. All animal experiments were carried out in compliance with and authorized by the Animal Care and Use Committee of the University of Science and Technology of China (USTC).

### Fertility test

Starting from 8 weeks of age, the fertility of wild-type (WT) and Prmt1-cKO male mice housed with fertility-proven adult WT females was examined as previously described. One male mouse was allowed to breed with at most two females in each cage. The pregnant females that had been plugged were separated and placed in isolated cages. The fertility test was performed for at least 6 months for each breeding pair.

### Tamoxifen injection scheme for inducible Cre-driver lines

For *in vivo* gene deletion (Ubc-CreERT2 and Ddx4-CreERT2) experiments, mice (Ubc-CreERT2-Prmt ^lox/lox^ and Ddx4-CreERT2-Prmt^lox/lox^) at various time intervals were injected with tamoxifen intraperitoneally (75mg/kg body weight). Tamoxifen (Sigma-Aldrich) was dissolved in corn oil at a concentration of 20 mg/ml by sonication at 60°C for 10 min or by shaking at 37°C overnight with protection by aluminum foil from the light, and stored at 4°C over the period of injection (−20°C for long-term storage). By tracking the Cre-mediated Loxp-recombination efficiency, tamoxifen injection was performed for three or four consecutive days as needed. During the time of injection and post-injection, the mice were scrutinized for adverse reactions.

### Histological analysis

The mice were sacrificed by cervical dislocation after isoflurane inhalation in a closed chamber. Seventy percent ethanol was sprayed on the ventral abdomen, and a V-shaped opening was made in the abdominopelvic cavity using sterile scissors and forceps. The epididymal fat was pulled out to locate the testes, dissected by using scissors and placed on a petri dish containing phosphate-buffered saline (PBS). The testes were isolated and fixed in Bouin's solution overnight on a rotator. The tissue was carefully positioned and embedded in paraffin and was subsequently sectioned into 5 μm thick slices using a Leica vibratome machine. The sections were deparaffinized and rehydrated sequentially in gradient concentrations of ethanol series, followed by hematoxylin and eosin (H&E) staining. After mounting the sections with neutral resins, images were captured by using a light microscope (MShot paired with MSX2 camera).

### Immunofluorescence

For immunofluorescence staining of paraffin-embedded tissues, the sections were dewaxed and rehydrated, followed by antigen retrieval through heat-induced epitope retrieval (HIER) (20,21). The rack of slides was placed in a vessel that contained boiled sodium citrate buffer (pH 6.0). The slides were exposed to HIER treatment for 30 min. Thereafter, the slides were subjected to immunofluorescence staining, starting by washing with 1× PBS (5 min each for three times), followed by permeabilization (0.2% Triton X-100 in 1× PBS) for 10 min at RT. After blocking, the sections were incubated with diluted (50% 1× PBS and 50% blocking buffer) primary antibody at 4°C overnight. The next day, the samples were rinsed in 1 × PBST (containing 0.1% Tween) three times, followed by a 60 min incubation at 37°C with a secondary antibody for visualization. The slides were dried down after DAPI staining, mounted using an antifade mounting medium and images were acquired on an inverted confocal fluorescence microscope (LSM980, Zeiss).

The following primary antibodies were used for immunofluorescence: anti-Prmt1 (1:200; M3008, Abmart), anti-ADMA [1:500; 13522S, Cell Signaling Technology (CST)], anti-MMA (1:500; 8711S, CST), anti-SDMA (1:500; 13222S, CST), anti-Sox9 (1:300; A19710, ABclonal Science), anti-Pcna (1:500; 60097-I-Ig, Proteintech®), and anti-Ki67 (1:500; GB111499, Servicebio), anti- Gfrα1 (1:40; AF560, R&D), anti-c-Kit (1:200; A0357, ABclonal Science), anti-Ddx4-Vasa (1:500; 51042–1-AP, Proteintech®), anti-GCNA (1:750; Ab82527, Abcam), anti-Plzf (1:350; 28319, Santa Cruz Biotechnology), anti-H4R3me2a (1:500; 667, PTM BIO) and anti-H4R3me2a (1:200; 39006, Active Motif).

### Whole-mount immunofluorescence

Testes were carefully isolated and decapsulated using fine-tipped scissors by cutting an incision in the *tunica albuginea* (fibrous sheet). The tubules were forced out with forceps by pressing, and the tunica was discarded. The tubules were pipetted up and down several times, then transferred to a 5 ml tube containing cold 1× PBS and allowed to sediment on ice. During sedimentation, the supernatant consisting of the cell debris and interstitial cells was removed carefully without dislodging the tubules, and fresh ice-cold 1× PBS was added and mixed by inversion. Subsequently, the supernatant was removed, and 4% PFA was added to the tubules for fixation for 4 hours on a rotator. After fixation, the fixative buffer was removed and the tubules were washed twice with 1× PBS, followed by dehydration using a methanol series. Subsequently, the tubules were hydrated in a methanol series and washed, followed by blocking for 1 h at RT. After blocking, the tubules were incubated in diluted primary antibodies at 4°C overnight. The next day, the tubules were rinsed in 1× PBST three times and incubated with secondary antibodies using the recommended dilutions for 1–2 h at RT in a black staining box. After incubation, the tubules were washed with 1× PBST twice and spread onto a microscopic slide by draining off the excess buffer. The tubules were mounted using an antifade mounting medium and covered with glass coverslips.

### RNA extraction and mRNA expression analysis using RT-qPCR

Total RNA was extracted from whole testes or somatic organs following standard protocols as described previously (22). In brief, the fresh tissue was homogenized in TRIzol reagent (1 ml per 50–100 mg tissue) supplemented with 3 mm RNase-free beads (Servicebio, Cat no G0203) using a homogenizer. The lysate was centrifuged and the supernatant was transferred to a fresh tube, followed by centrifugation upon chloroform addition. The upper phase (clear-aqueous) was transferred to a fresh tube and an equal volume of 70% ethanol was added. Subsequently, a kit (Qiagen-74104) protocol was followed and treated by DNase I (Invitrogen™-18068015). The quality and yield were assessed through NanoPhotometer® N50 (Implen) and Qubit assay (Thermo Fisher Scientific). First-strand cDNA reverse transcription was carried out using a PrimeScript RT reagent kit (Vazyme, R302-1) with gDNA Eraser to eradicate DNA contamination. Real-time RT-PCR was performed using a Hieff® qPCR SYBR Green Master Mix (No Rox) on a Q2000B Real-Time PCR System. Relative gene expression was analysed by the method of 2^−ΔΔCT^ as described by Kenneth J. Livak (23). All qPCR primers are listed in Supplementary Table S1. Data are presented as mean ± SEM unless otherwise stated. Student's *t-test* was employed to examine group differences. *P* < 0.05 was deemed statistically significant.

### Isolation of spermatogonia by fluorescence-activated cell sorting (FACS)

The testes from the heterozygous Ddx4-GFP-CreERT2-Prmt1^+/lox^ and Prmt1-dKO (Ddx4-GFP-CreERT2-Prmt1^lox/lox^ male mice at P7 were decapsulated in a petri dish containing Dulbecco's Modified Eagle Medium (DMEM). Tubules were dispersed using sterile forceps and digested into a single-cell suspension by collagenase and trypsin enzymes in DMEM medium. Briefly, the tubules were transferred to fresh collagenase buffer and incubated at 37°C for 5 min, followed by rinsing twice or three times with 1× Krebs–Henseleit solution (Krebs) to remove interstitial somatic cells. The sedimented tubules were transferred to a 60 mm dish and delicately chopped using sterile forceps, which were further digested into single cells in 10 ml trypsin buffer by incubation at 37°C for 10 min. The digestion was quenched by 2% FBS.

For flow sorting of spermatogonial cells, a new template for FACSCalibur™ was documented with a density plot to set up the machine and gather data. To reduce events on the axes that result in a single cell population, a dot plot with FSC (forward scatter) for the X-axis and SSC (side scatter) for the Y-axis was adjusted. The fluorophore signal from GFP-positive spermatogonia was gated to determine the fluorophore laser settings.

### Modified Smart-seq2 library construction for low-input cell samples

Total RNA was extracted from a limited amount of FACS-sorted spermatogonial cells (∼1 × 10^4^ in total for each sample) using a MACHEREY-NAGEL micro-kit (MACHEREY-NAGEL, Germany). RNA quality and concentration were monitored by a Qubit dsDNA assay kit (Thermo Fisher Scientific, USA) and qPCR analyses. The full-length mRNA library was prepared using an *in-house* optimized Smart-seq2 protocol (24). Briefly, ∼10 ng of total RNA for each sample was utilized for first-strand cDNA reverse transcription in a 10 μl RT buffer containing SuperScript III RTase (100 U), RNase inhibitor (10 U), dNTP mix (10 mM each), dCTP (10 mM), SS III first-strand buffer (1×), DTT (5 mM), betaine (1 M), MgCl_2_ (6 mM), TSO (1 μM) and oligodT30VN (10 μM). The RT reaction was performed at 60°C for 50 min. The full-length cDNA was subsequently amplified through semi-suppressive PCR for 15 cycles in a buffer containing 10 μl of first-strand cDNA, 12.5 μl KAPA HiFi HotStart ReadyMix (1×), ISPCR primers (0.1 μM), and nuclease-free water. The amplified full-length DNA library was purified to get rid of <500 bp fragments and other contaminants using Hieff NGS® DNA Selection Beads (Yeasen) following the manufacturer's protocol. 1 ng of purified DNA was exploited for library preparation using TruePrep® DNA Library Prep Kit V2 for Illumina (Vazyme). The final DNA library cleanup was carried out by double-side bead selection (0.6×/0.3×) to extract library DNA in sizes ranging from 350 to 550 bp as measured by Bioanalyzer 2100 (Agilent), and was ultimately subjected to library sequencing by Novaseq 6000 (PE150 mode).

### RNA-seq library preparation

Total RNA was extracted from whole testis using the E.Z.N.A.® MicroElute® Total RNA Kit following the manufacturer's protocol. DNase I-treated total RNA samples were subjected to polyA mRNA isolation, followed by heat-induced mRNA fragmentation prior to first-strand cDNA reverse transcription using randomized hexamer primers. After second-strand DNA synthesis, the double-strand DNA was end-repaired prior to dA-tailing, followed by Y adaptor ligation, according to standard protocols (24).

### RNA-seq/modified Smart-seq2 data analysis

The raw data were subjected to quality-control using FastQC (version 0.11.9), followed by fastp (version 0.23.2) to remove the unwanted and low-quality reads. The paired-end clean reads were mapped to the mouse reference genome (mm10) using STAR (version 2.7.6a). The mapping files containing the matching reads were run through RSEM to calculate the expression values for genes and transcripts, as well as the raw read counts. Genes with averaged TPM ≥ 1 were deemed as expressed genes (∼16 000 genes detected per sample). The DESeq2R package was employed to analyze the differential expression differences between the control group and the experimental group (cutoff: fold change ≥ 2; adj-*P*-value ≤ 0.05). GO enrichment was performed by the DAVID database (25).

For alternative splicing analyses, the rMATS (version 4.1.2) software was run for the fastq file with the following parameters: – Read length 150 –variable-read-length. The false discovery rate (FDR)≤0.05 was categorized as differentially alternative splicing events. The rmats2sashimiplot was used to convert the rMATS output into a Sashimi plot.

### Histone isolation

For histone isolation, we followed a standard protocol (26). In short, the tissue was transferred to a Dounce homogenizer after weighing and homogenized into a single-cell suspension (smaller clumps) in TEB buffer (1 ml/200 mg tissue) with 50–60 strokes. The tissue pellet was resuspended in 3 volumes of acidic buffer (0.5N HCl + 10% glycerol) and incubated for 30 min on ice with rotation at 4°C, followed by centrifugation at 12 000 rpm for 5 min. The supernatant was transferred to a fresh tube and processed for Trichloroacetic acid (TCA) precipitation. The histones were recovered by centrifugation for 10 min at 12 000 rpm. After supernatant removal, the pellet was sequentially washed with ice-cold acetone containing 0.05% HCl, and three times with ice-cold acetone. After successive washing the pellet was dried at −20°C overnight, then dissolved in water and stored at −20°C.

### Immunoblot and protein band quantification

The tissues were freshly collected in liquid nitrogen for snap freezing. Samples were homogenized in ice-cold RIPA buffer using an electric homogenizer (Servicebio-G0203). The homogenized sample was centrifuged and the supernatant was transferred to a fresh tube. Pierce™ BCA Protein Assay Kit (Thermo Scientific™) was used to quantify the protein concentration. Both semi-dry and wet-transfer methods were used and the membranes were blocked (5% skim milk) for 60 min at RT, followed by primary antibody (diluted in 5% milk) incubation at 4°C overnight. The images were obtained from the membrane with the clinix-science instrument coupled with a CCD camera. When needed, the membrane was stripped in stripping buffer (pH 2.2) before probing with another antibody. ImageJ (NIH) was used to measure the intensity of protein bands for densitometric analysis. In a nutshell, the whole protein band's ROI was chosen, and integrated intensity was assessed using the set measurement menu. The integrated intensities values for the same size selection were also applied to areas below and above the specific band used as background.

The following primary antibodies were used for immunoblot: anti-Prmt1 (1:2000; M3008, Abmart), anti-ADMA (1:1000; 13522S, CST), anti-MMA (1:1000; 8711S, CST), anti-SDMA (1:1000; 13222S, CST), anti-Prmt2 (1:500; MG443642, Abmart), anti-Carm1 (1:500; 200135, ZEN BIO), anti-Prmt5 (1:1000; 381019, ZEN BIO), anti-Prmt6 (1:1000; 383079, ZEN BIO), anti-Prmt7 (1:3000; R25450, ZEN BIO), anti-c-Kit (1:1000; A0357, ABclonal Science), anti-H3 (1:1500; ET1701-64, HUABIO), anti-Gapdh (1:30 000; 60004-1-Ig, Proteintech®), anti-H4R3me2a (1:1500; 667, PTM BIO), anti-H4R3me2a (1:500; 39006, Active Motif) and H3R2me2a (1:1000; A-3714, EPIGENTEK).

### Cleavage under targets & tagmentation (CUT&Tag) assay

To decipher the chromatin localization landscape of histone modification markers, cleavage under targets & tagmentation (CUT&Tag) was carried out as previously described (27). In brief, the testes from two time points were collected, including spermatogonia-enriched (P7) and spermatocytes (P14). After removing the tunica albuginea, the seminiferous tubules were digested with Collagenase IV briefly to eliminate the somatic interstitial cells. The pure tubules were digested by trypsin into homogeneous single-cell suspension, with filtering through 40 μm cell strainers to eliminate Sertoli cell contamination, as described previously (28,29). For CUT&Tag, the single-cell capture, antibody incubation and Tn5 transposon activation were carried out according to the procedures as described in Hieff NGS® G-Type In-Situ DNA Binding Profiling Library Prep Kit (Yeasen) following the manufacturer's instructions. For each sample, briefly, an averaged total of ∼50 000 single cells were allowed to bind the ConA beads. The primary antibody was incubated with the beads-cell suspension on a rotator at 4°C overnight. The next day, the secondary antibody was added and incubated for 60 min at RT, followed by incubation with pA/G-Tn5 transposome and tagmentation in activating buffer supplemented with magnesium. The adaptor-ligated DNA fragments were extracted and PCR-amplified using full-length index primers. The amplified product was purified using Hieff NGS® DNA Selection Beads (Yeasen), and the quantity and quality of the DNA library were examined through Qubit™ 4 Fluorometer and Bioanalyzer 2100 (Invitrogen), respectively. The libraries were sequenced by NovaSeq 6000 (PE150).

The following primary antibodies were used for CUT&Tag: anti-H4R3me2a (1:100; 39006, Active Motif), anti-H4R3me1 (1:100; NB21-2011SS, Novus Biologicals), anti-H4R3me2s (1:200; 61988, Active Motif), anti-H3R2me2a (1:100; A-3714, EPIGENTEK), anti- H3R8me2a (1:100; NB21-1062, Novus Biologicals), anti-H3K4me3 (1:100; A2357, ABclonal Science) and anti-H3K27ac (1:100; A7253, ABclonal Science).

### CUT&Tag data processing and alternative splicing analysis

The peaks were called using MACS2 (version 2.1.0). The bamCoverage command in deeptools (version 3.5.1) was used to convert the bam file to a bw file that has been normalized by CPM. CUT&Tag densities were visualized by Integrative Genomic viewer (IGV). The deeptools commands multiBigwigSummary and plotCorrelation were used to calculate the sample correlation. ComputeMatrix and plotProfile (plotHeatmap) commands were used for assessing the peak distributions of histone modifications at TSS, TES, and CGI.

To visualize CUT&Tag signal pattern for both constitutive and skipped exons, we first acquired the coordinates (exonStart_0base, exonEnd) of the target skipped exons. In such case, the adjacent up and downstream exons were termed constitutive. The surrounding region of the splice junction was extended 100 bp to the 5′ and 3′ ends following normalization, the CUT&Tag signal intensity was calculated, and the Student's *t-test* was used to compare the statistic difference for both sides of exons, as well as the regions between alternative and constitutive exons. To account for the difference in the signal distribution of histones on exons and introns, we excluded the influence of TSS by removing first exon and setting the minimum length for intron 500 bp, whereas for exon we set minimum length of 200 bp.

### TUNEL assay

The TUNEL assay was performed using the Vazyme FITC apoptosis kit (A111) following the manufacturer's instructions with some minor modifications. Briefly, the sections were permeabilized using 0.3% Triton X-100. After equilibrating the sections, the TdT reaction mixture was added and incubated for 60 min at 37°C. Afterward, the sections were incubated for 10 min with DAPI (1:1000) for nucleus counterstaining. Following the final wash, the slides were dried with Kimwipes and mounted with antifade mounting media before storage at 4°C in a black box until microscopic imaging.

### Statistical analyses

All experiments in this study were conducted at least in biological triplicates unless otherwise stated. All statistical analyses were performed using Prism 8 (GraphPad software). Unless otherwise stated, statistical significance was determined using the Student's *t*-test. *P* values of <0.05 were deemed statistically significant. *, **, *** and **** represent *P* < 0.05, *P* < 0.01, *P* < 0.001 and *P* < 0.0001, respectively.

## RESULTS

### Prmt1 is highly expressed and predominantly localized to the nuclei in spermatogonia of mouse testes

**Figure 1.**
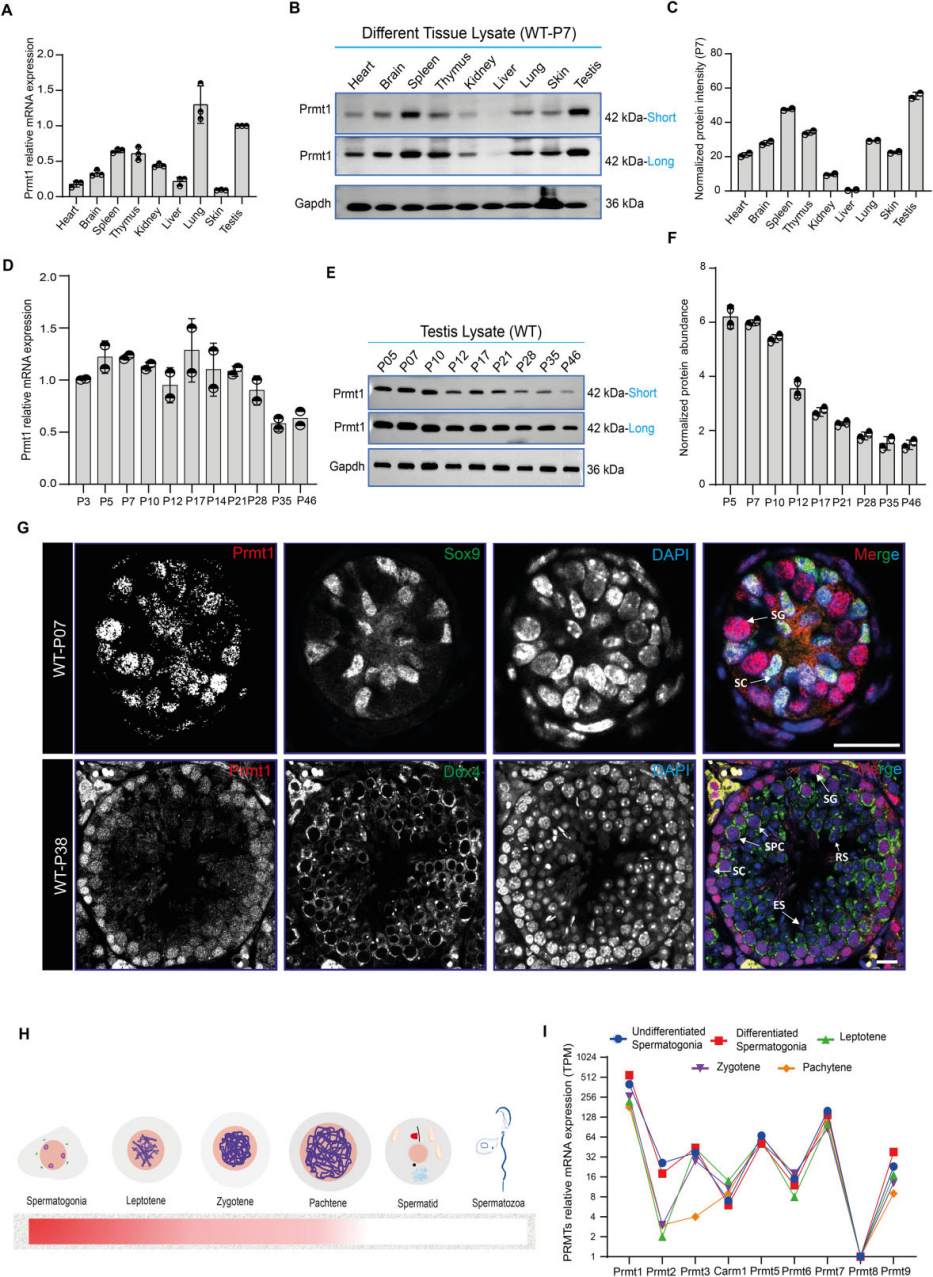
Prmt1 is highly expressed and predominantly localized to the nucleus in spermatogonia in mouse testes. (**A**) Relative quantification of Prmt1 mRNA expression levels across different tissues in mice (n = 3 mice per genotype). (**B**) Immunoblotting of Prmt1 in wild-type (WT) mouse tissues at postnatal day 7 (P7). Gapdh served as a loading control. **(C**) Quantitative data show normalized protein intensity of Prmt1 at P7 in WT mice from ‘B’ (*n* = 2). (**D**) Relative mRNA levels of Prmt1 in WT developing testes (*n* = 2). (**E**) Immunoblotting of Prmt1 in WT developing testes (*n* = 2). (**F**) Quantitation of normalized protein intensity for Prmt1 in WT mouse testes from ‘E’ (*n* = 2). (**G**) Immunostaining of Prmt1 and Sox9 in testicular sections from WT mice at P7 and P38. Scale bars, 20 μm. (**H**) A schematic illustration showing the expression of Prmt1 in WT testis. Pink color represents the nuclear localization of Prmt1 protein. (**I**) mRNA expression of PRMT members at single-cell levels in different developmental stages of germ cells in P15 juvenile mice (68), TPM, transcript per million.

We first assessed the multi-organ mRNA expression levels of Prmt1 in mice. qPCR assays showed that the highest Prmt1 mRNA levels were detected in mouse lung and testis (Figure 1A). However, at the protein level, Prmt1 is most abundantly present in the testis compared with other somatic tissues at postnatal day 7 (P7) (spermatogonia) or P42 (when the first wave of spermatogenesis is completed) (Figure 1B, C and Supplementary Figure S1A, B), suggesting Prmt1 is likely subject to post-transcriptional regulation. The male germline development is strictly time-dependent, i.e. the specific type/stage of germ cells proceeds at defined time points (30). We thus examined the expression of Prmt1 during postnatal testicular development. The qPCR assay showed relatively comparable mRNA expression levels for Prmt1 (Figure 1D), whereas Prmt1 protein displayed higher levels around P5∼P10 and decreased apparently thereafter (Figure 1E, F), presumably implying that Prmt1 mRNA is subject to post-transcriptional regulation as well in the germline, and that Prmt1 is most likely expressed in spermatogonial cells, since the highest proportion of spermatogonial cells are present in the younger testis (<P10). Therefore, we next conducted immunofluorescent staining (IF) on cryosections of mouse testes at various developmental time points (Figure 1G and Supplementary Figure S1C) as well as the immunohistochemistry (IHC) staining on paraffin-embedded testicular sections (Supplementary Figure S1D). Co-staining with Sertoli cell-specific marker (Sox9) and the germline-specific Ddx4 marker, demonstrated that the Prmt1 protein is predominantly expressed in the spermatogonial population (undifferentiated & differentiated), with markedly declined levels detectable at later stages of germ cells (e.g. meiotic spermatocytes or haploid spermatids) (Supplementary Figure S1C–E). Noticeably, the Prmt1 protein is largely distributed within the nuclei of spermatogonia during postnatal germ cell development (Figure 1G-H and Supplementary Figure S1C–E). We further evaluated Prmt1 mRNA expression at the single-cell level using published testicular single-cell RNA-seq datasets (Supplementary Figure S1F, G). In agreement with findings above, the Prmt1 mRNA levels are highest in spermatogonia when compared with other types of germ cells in juvenile or adult mouse testes. Noteworthily, Prmt1 displays the highest expression levels among the PRMTs (Prmt1∼9) based on the single-cell RNA-seq analyses in P15 testes (Figure 1I), presumably implying that it has non-redundant function with other PRMT family members during germline development. Together, this evidence validated that Prmt1 is strongly enriched in the nuclei of spermatogonial cells, but weakly in the nuclei of spermatocytes, within mouse testes.

### Prmt1 is indispensable for spermatogenesis and male fertility

**Figure 2.**
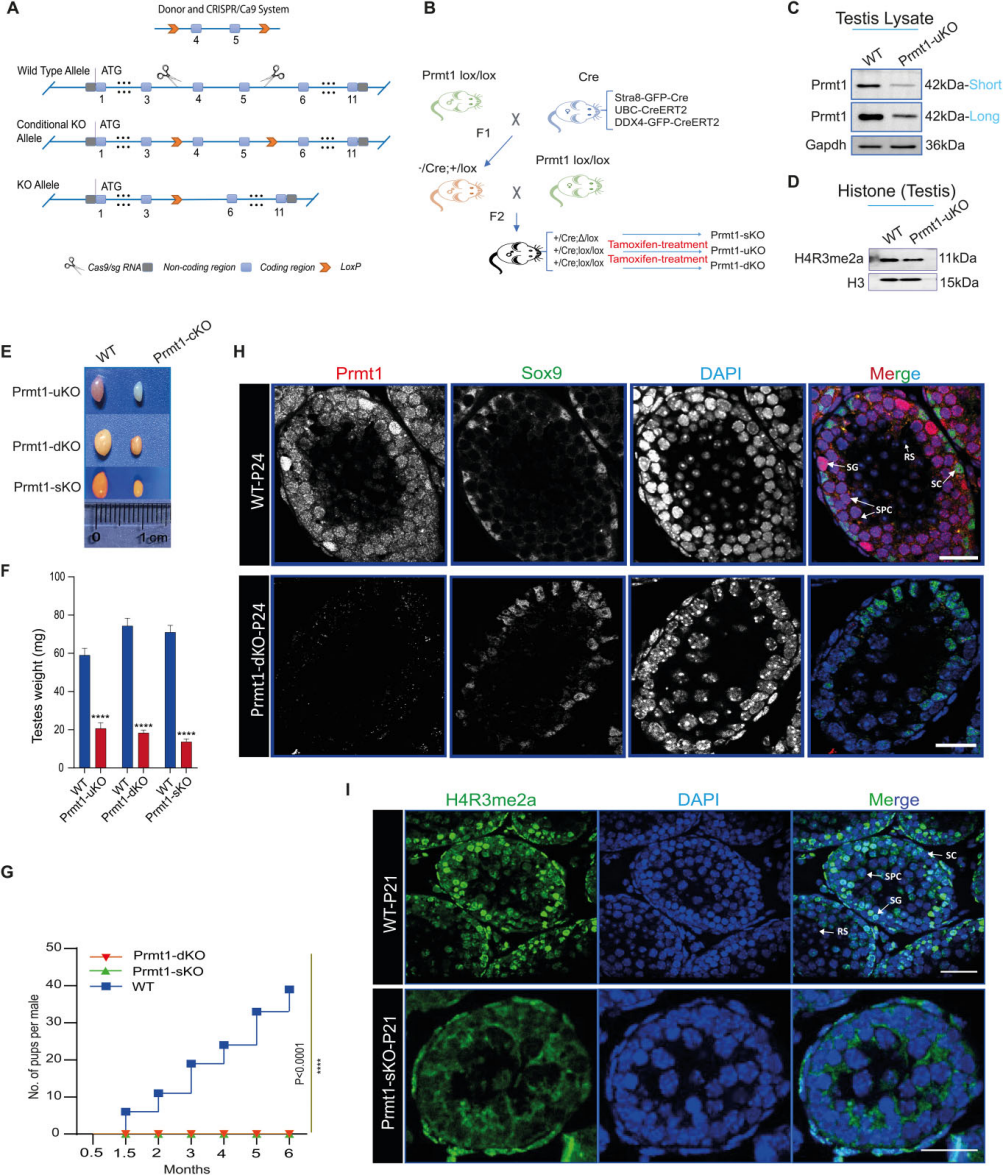
Prmt1 is indispensable for spermatogenesis and male fertility. (**A**) Schematic diagram depicting the targeting strategy for Prmt1 conditional knockout construct. (**B**) Schematic representation of breeding scheme by crossing Prmt1^lox/lox^ with the three Cre-lines. (**C**) Immunoblotting of Prmt1 in WT and Prmt1-uKO mice at P42. Gapdh served as a loading control. (**D**) Immunoblotting of Prmt1 substrate (H4R3me2a) in WT and Prmt1-uKO testes. H3 served as a loading control. (**E**) Gross morphology of adult mouse testis from Prmt1-uKO, Prmt1-dKO, and Prmt1-sKO mice as compared with WT mice. (**F**) Averaged testicular weights derived from Prmt1-uKO, Prmt1-dKO, and Prmt1-sKO mice, as compared with those in WT. Error bars indicate SEM. ****P< 0.0001. **(G)** Fertility test for Prmt1-dKO, Prmt1-sKO and age-matched WT males for 6 months. The X-axis represents the time periods during mating; Y-axis indicates the number of pups. Error bars indicate SEM. ****P< 0.0001. (**H**) Immunostaining of Prmt1 and Sox9 in testicular sections from WT and Prmt1-dKO mice at P24. Scale bars, 20 μm. (**I**) Immunostaining of H4R3me2a in testicular sections from WT and Prmt1-sKO mice at P21. Scale bars, 20 μm.

To study the role of *Prmt1* in germline *in vivo*, we generated the Cre-inducible *Prmt1^loxp^* mouse allele since global ablation of *Prmt1* led to embryonic lethality (31). There is a total of 14 predicted Prmt1 transcripts (Prmt1-201∼214) in the Ensembl mouse database (GRCm39, GCA_000001635.9), of which the Prmt1-202 (ENSMUST00000107843.11) encodes the longest protein isoform consisting of closely juxtaposed exons 4 and 5 conserved across all predicted transcripts. Moreover, ablation of exons 4 and 5 presumably elicits frame-shift mutation causing aborted function of Prmt1 *in vivo*. Therefore, exons 4 and 5 were selected for *Loxp* cassette flanking to produce the *Prmt1^loxp^* allele (Figure 2A). Unlike somatic cells, germ cell development is not synchronized, but rather, is genetically programmed and spatiotemporally regulated. We thus exploited three Cre lines to cross with *Prmt1^loxp/lox^* mice, including *Stra8-Cre* (specifically activated in spermatogonia starting from P3 testis), *Ubc-CreERT2* (Tamoxifen [Tam]-inducible in all cell types), and *Ddx4-CreERT2*, which is *in-house* generated, tamoxifen-inducible specifically driven by *Ddx4* promoter in germ cells (see Materials and Methods). These lines thereby provided powerful genetic tools to dissect the time-dependent, and the stage-specific roles of Prmt1 in the mouse germline. Through two generations of crossings between Cre lines and *Prmt1^loxp/lox^* alleles, we obtained the *Stra8-Cre; Prmt1^loxp/lox^* (hereafter referred to as Prmt1-sKO), *Ubc-CreERT2; Prmt1^loxp/lox^* (Prmt1-uKO, following Tam injection) and *Ddx4-CreERT2; Prmt1^loxp/lox^* (Prmt1-dKO, following Tam injection), respectively (Figure 2B).

To examine the KO efficiency of *Loxp* cassettes, we injected Tam into both WT and Prmt1-uKO males for three consecutive days starting from P12, and sacrificed them at day 7 of post-tam injection. Immunoblotting assays on the testicular lysates from the seminiferous tubules with antibodies against Prmt1 or its known histone methyl substrate—H4R3me2a, verified that the levels of both Prmt1 protein and H4R3me2a were significantly declined in the Prmt1-uKO testes, as compared with WT testes (Figure 2C, D). Next, we characterized the phenotypic outcome upon *Prmt1* inactivation. As seen in Figure 2E, F, the testicular sizes in all Prmt1-sKO/uKO/dKO males apparently dropped down to by less than one-fifth of those in WT males. In agreement with this, >5-month fertility test demonstrated that all KO males were completely sterile, signifying that spermatogenesis was disrupted in *Prmt1-null* mouse testes (Figure 2G). Consistently, immunofluorescent staining revealed that the signals for both Prmt1 and H4R3me2a were largely demolished from Prmt1-dKO and Prmt1-sKO testes (Figure 2H, I). Taken together, these data suggest Prmt1 is indispensable for testicular germ cell development and male fertility.

### Prmt1 modulates the protein substrate arginine methylome in the germline distinct from somatic cells

**Figure 3.**
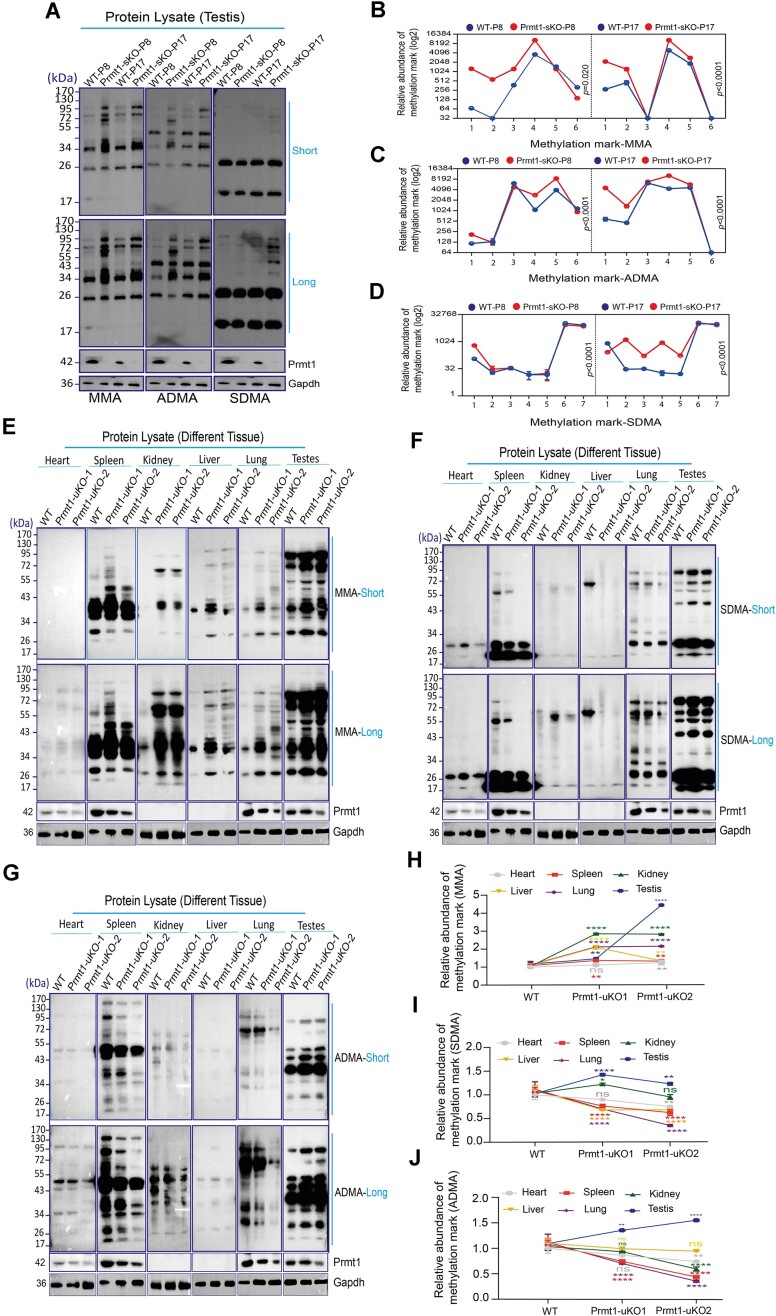
Prmt1 modulates the substrate arginine methylome in the germline distinct from somatic cells. (**A**) Immunoblotting of MMA, ADMA and SDMA in WT and Prmt1-sKO mice at P8 and P17. Short, short exposure; long, longer exposure. Gapdh serves as an internal control. The marker sizes are labelled (KDa). (**B–D**) The line charts depict the relative expression intensities for the visible bands for MMA, ADMA and SDMA marks with normalization (log_2_), respectively. Each dot represents the relative intensity for each visible band from the top to the bottom in each sample, as calculated by densitometric analysis (using ImageJ). Student's *t-test*, mean ± SEM (*n* = 2). (**E**) Immunoblotting of MMA in WT and Prmt1-uKO mice at indicated time (Supplementary Figure S2A). (**F**) Immunoblotting of SDMA in WT and Prmt1-uKO mice at indicated time (Supplementary Figure S2A). (**G**) Immunoblotting of ADMA in WT and Prmt1-uKO mice at indicated time (Supplementary Figure S2A). (**H–J**) Quantitation of normalized band densitometries for MMA, SDMA and ADMA immunoblots from ‘E–G’, respectively, as described in (B–D). Note the same blots for Prmt1 and Gapdh loading controls were used since they were derived from the same samples. *P< 0.05, **P< 0.01, ***P< 0.001 and ****P< 0.0001; mean ± SEM, and *n* = 3.

As the primary Type-I arginine methyltransferase, loss of Prmt1 activity spurred an eminent increase of global levels for both MMA and SDMA by other PRMT members (32). Since the Prmt1 protein is present in the nuclei of both the mitotic spermatogonia and meiotic spermatocytes, as shown above (Figure 1), we next interrogated how the substrate methylarginine cross-talks upon *Prmt1* depletion in testicular germ cells. To this end, we performed the immunoblotting on the Prmt1-sKO testicular lysates collected at P8 (dominated by spermatogonia) and P17 (spermatocytes) with three pan-methylarginine antibodies—MMA, ADMA and SDMA. These antibodies were generated using a mixture of short stretches of peptides containing methylated arginine residues within consensus motifs, and immune-react with the corresponding methylarginine (33). Consistent with prior studies in somatic cells, we found a dramatic increase in MMA levels and a moderate enhancement of SDMA methylation (Figure 3A–D), suggesting that there is a substrate cross-talk between Prmt1 and other PRMT members in testes. However, surprisingly, we discovered that the ADMA levels were significantly up-regulated as well, especially in P8 testes. Notably, theh increased signal comprises novel bands that were not seen in the WT testes, in addition to the shared bands present in both WT and Prmt1-sKO testes, hinting that Prmt1 likely orchestrates a distinctive network of substrate ADMA methylation in the germline.

To test whether the elevated ADMA levels are restricted to testes as opposed to other primary tissues, we next compared the expression levels of all three methylarginine marks (MMA, ADMA, and SDMA) between testes and a select panel of somatic organs (heart, spleen, kidney, liver and lung) by employing the ubiquitous expression of the Ubc-Cre mouse line (Prmt1-uKO). We carried out the Tam injection for three consecutive days and collected the tissues on the fourth and seventh day post-Tam injection (Supplementary Figure S2A). In line with the high levels of Prmt1 protein in the spleen and the testis, both organs exhibited higher levels of all three types of substrate arginine methylation than other tissues (Figure 3E–G). Consistently, we observed that the MMA levels were most drastically up-regulated upon loss of Prmt1 activity in all the testis and somatic tissues (Figure 3E and H). The SDMA levels were also markedly enhanced upon Prmt1 KO in testis (Figure 3F and I). Not surprisingly, the ADMA levels dropped significantly in the somatic organs upon *Prmt1* loss (Figure 3G and J). Nevertheless, in agreement with observation in Prmt1-sKO testes, the ADMA marks were unambiguously elevated in *Prmt1-null* testes, which is opposite to the observation in soma (Figure 3C and J). Therefore, Prmt1 KO reshaped the global substrate methylation dynamics for all three types of methylarginine, which is in sharp contrast to the phenomenon observed in somatic organs.

Prmt1 is ubiquitously expressed in all tissues. We further found that even in the adult mice, global KO caused rapid mouse lethality (9–15 days following Tam injection) in the Prmt1-uKO line (Supplementary Figure S2B). To track the dynamics of substrate methylation as well as to exclude the impact of interstitial cells seen in Prmt1-uKO, we conducted a time-course measurement of all three methylarginines in the Tam-inducible Prmt1-dKO testes, at four post-Tam injection time points: P16, P18, P20 and P22 (Supplementary Figure S2C). In support of the findings above, while the substrate SDMA levels were less strong compared with the other MMA and ADMA marks, all three methylarginine levels were unambiguously enhanced upon tamoxifen-induced *Prmt1* deletion in the Prmt1-dKO testes (Supplementary Figure S2D–G). Of note, at P22, all three types of methylation levels were slightly decreased compared with those at P20, which presumably resulted from the loss of germ cells during long-term Tam-induced *Prmt1* KO. To explore this hypothesis, we performed immunofluorescence staining with the germline-specific marker, GCNA, and counted the various types of germ cells in mouse testes. This revealed that, whereas there appeared to be no loss of germ cells at P16, the numbers of meiotic and haploid spermatids were significantly reduced at P22 (Supplementary Figure S2H–K), which accounted for the reduced substrate methylation, in Prmt1-dKO testes at P22. Altogether, these data suggest that Prmt1 activity alter an intricate substrate cross-talk both among three methylarginine types and within Type I asymmetric dimethylation in the germline.

### Prmt1 depletion evokes an intrinsic interplay with Prmt2, Prmt5 and Prmt6 responsible for the enhanced SDMA and ADMA methylation in the testis

**Figure 4.**
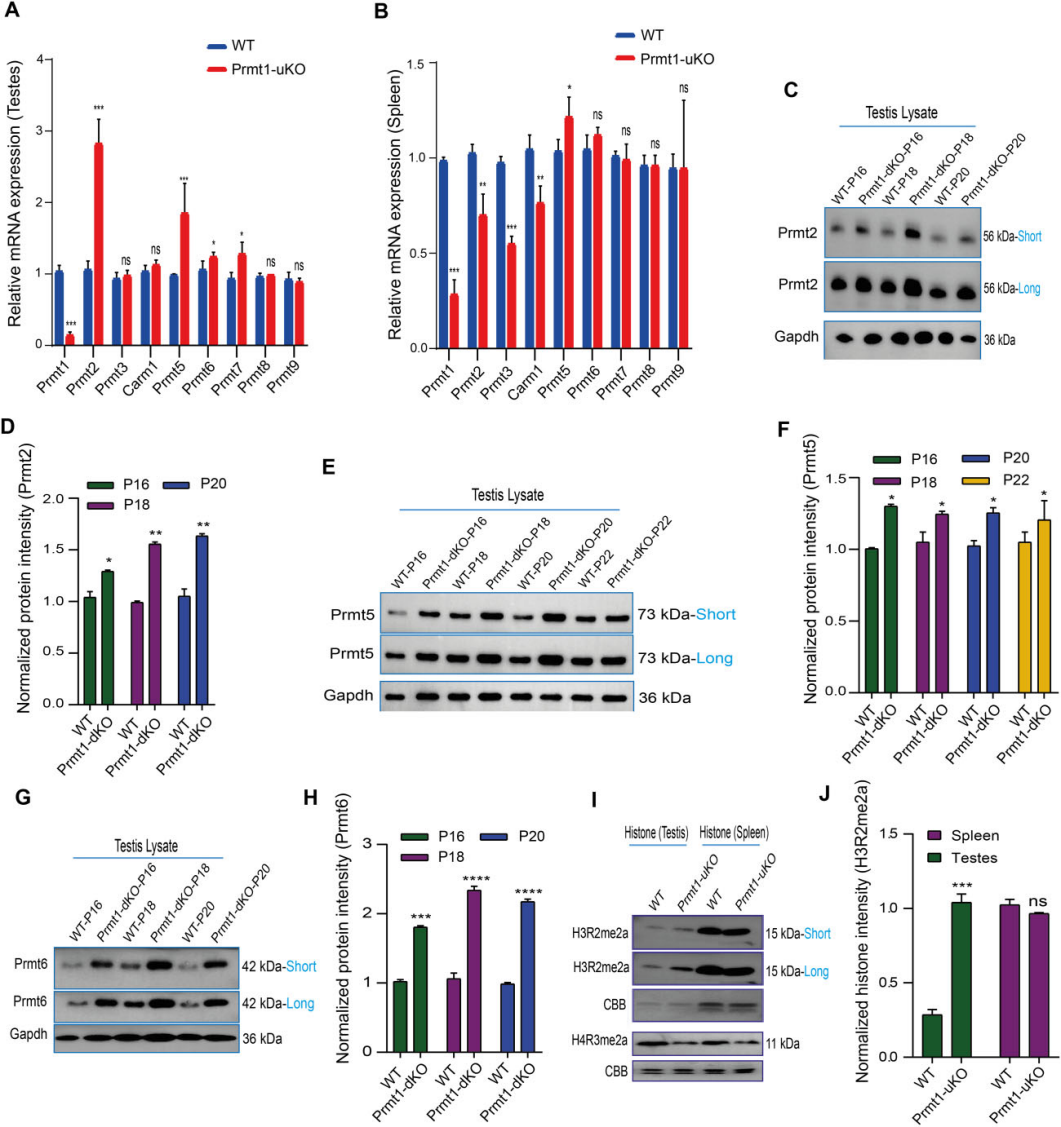
Prmt1 depletion evokes an intrinsic compensatory increase of Prmt2 and Prmt6 responsible for the enhanced ADMA substrate methylation in testis. (**A**) Relative mRNA expression of PRMTs in testis from WT and Prmt1-uKO mice at indicated time (Supplementary Figure S3A), (*n* = 3). *P< 0.05, **P< 0.01, ***P< 0.001 and ****P< 0.0001, Student's *t*-test. (**B**) Relative mRNA expression of PRMTs in spleen from WT and Prmt1-uKO mice at indicated time (Supplementary Figure S3A) (*n* = 3). *P< 0.05, **P< 0.01, ***P< 0.001 and ****P< 0.0001, Student's *t*-test. (**C**) Immunoblotting of Prmt2 in WT and Prmt1-dKO mice at indicated time (Supplementary Figure S2C). Gapdh served as a loading control. (**D**) Quantification of normalized protein band intensities for ‘C’ (*n* = 2). *P< 0.05 and **P< 0.01. (**E**) Immunoblotting of Prmt5 in testis from WT and Prmt1-dKO mice at indicated time (Supplementary Figure S2C). (**F**) Quantification of normalized protein band intensities for ‘E’ (*n* = 2). *P< 0.05. (**G**) Immunoblotting of Prmt6 in WT and Prmt1-dKO mice at indicated time (Supplementary Figure S2C). (**H**) Quantification of normalized protein band intensities for ‘G’ (*n* = 2). ***P< 0.001 and ****P< 0.0001. (**I**) Immunoblotting of H3R2me2a, H4R3me2a in WT and Prmt1-uKO mice at indicated time (Supplementary Figure S3A). Staining by Coomassie brilliant blue (CBB) served as a loading control. (**J**) Quantification of normalized protein band intensities for ‘I’ (*n* = 2). ***P< 0.001.

The significantly increased levels of ADMA upon loss of Prmt1 activity cannot be explained by the traditional notion that Prmt1 is primarily for ADMA deposition (Figure 3G), which led us to speculate that there might be compensatory effects attempted by other Type-I PRMTs when the pre-defined ADMA marks by Prmt1 are unmasked upon *Prmt1* ablation. To test this hypothesis, we performed the qPCR analyses for PRMT family members using the testis and spleen tissues derived from WT and Prmt1-uKO mice (Figure 4A and Supplementary Figure S3A). While the mRNA levels of Prmt1 were significantly down-regulated as expected, the levels for asymmetric arginine methyltransferase Prmt2/6/7, as well as the symmetric arginine methyltransferase Prmt5, were all markedly up-regulated, albeit with varied levels, in the Prmt1-uKO testes (Figure 4A). By comparison, in the spleen tissue, this phenomenon was not observed except for Prmt5 (Figure 4B). Notably, Carm1 mRNA levels were not altered in the testis, but rather decreased in the spleen. At the protein level, immunoblot analysis showed that the Prmt2/5/6 members were overtly up-regulated except for Carm1 in the Prmt1-dKO testes during the time-course of Tam induction (Figure 4C-H and Supplementary Figure S2C). Fluorescent immunostaining with Prmt5 revealed a sharp difference between Prmt1-dKO and WT testes (Supplementary Figure S3B). The dynamic expression of Prmt5 was abolished by Prmt1 loss. When *Prmt1* was deleted, Prmt5 is predominantly restricted in the nuclei as opposed to that in P22-WT testes, where Prmt5 is distributed in the cytoplasm of spermatocytes and round spermatids (Supplementary Figure S3B). At the same exposure time, higher Prmt5 expression levels were observed in the Prmt1-dKO testes (Figure 4E, F). We also examined the expression levels of the well-known Prmt6 substrate, H3R2me2a, using acid-purified histone proteins, and found that this methyl mark was indeed prominently up-regulated in the Prmt1-uKO testes (Figure 4I, J), but not in the Prmt1-uKO spleen. In contrast, although there were slight increases for both Prmt4 (Carm1) and Prmt7 upon Prmt1 loss, statistical analyses overall revealed comparable levels between the WT and Prmt1-dKO testes (Supplementary Figure S3C–H). Therefore, the enhanced levels for MMA, SDMA and ADMA in the *Prmt1-null* germ cells were likely achieved through Prmt2, Prmt5 and Prmt6. Interestingly, previous studies demonstrated that in the MEF cells, Prmt1 loss induced the compensatory elevation of Prmt6/7, concomitant with ADMA declining during early days of treatment, which is opposed to the phenomenon in germ cells (32). Together, these data prompt that there exists a cell-autonomous cross-talk among Type I and II enzymes (Prmt1/2/5/6) uniquely in germ cells, but not present in soma.

### Prmt1 is required to maintain the developmental trajectory of the spermatogonial population through the first wave of spermatogenesis and in adult mouse testes

**Figure 5.**
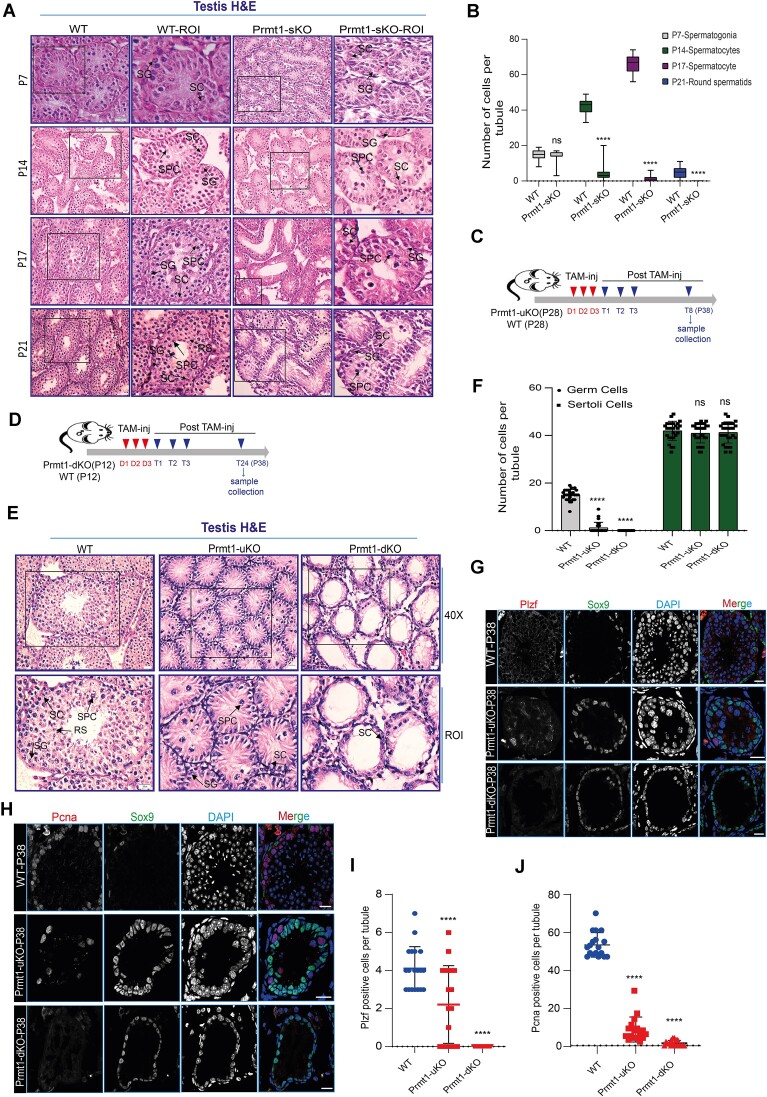
Prmt1 is required to maintain the developmental homeostasis of spermatogonial population through the first wave of spermatogenesis and in adult mouse testes. (**A**) H&E staining of testes from Prmt1-sKO (P7, P14, P17 and P21) and age-matched WT mice (ROI, region of interest). Note that apparent germ cell loss was observed in testes from P14 onwards. SG-spermatogonia, SPC-spermatocyte, SC-Sertoli cell and RS-round spermatids; Scale bar, 20 μm. (**B**) Quantification of the numbers for spermatogonia, spermatocytes, and round spermatids per cross-section of the tubule. ****P< 0.0001. (**C**) Tamoxifen injection and tissue collection scheme for Prmt1-uKO mice. (**D**) Tamoxifen injection and tissue collection scheme for Prmt1-dKO mice. (**E**) H&E staining of adult testicular sections from WT, Prmt1-uKO, and Prmt1-dKO mice. Note that the spermatogonia were depleted from the tubules. SG-spermatogonia, SPC-spermatocyte, SC-Sertoli cell and RS-round spermatids; Scale bar, 20 μm. (**F**) Quantification of the numbers of germ cells from ‘E’. ****P< 0.0001, Student's *t*-test, mean ± SEM (*n* = 3). (**G**) Immunostaining by Plzf and Sox9 in testicular sections from WT, Prmt1-uKO and Prmt1-dKO mice at indicated times. Scale bars, 20 μm. (**H**) Immunostaining by Pcna and Sox9 in testicular sections from WT, Prmt1-uKO and Prmt1-dKO mice at indicated times. Scale bars, 20 μm. (**I, J**) Quantification of the numbers of Plzf- and Pcna-positive germ cells; ***P< 0.001 and ****P< 0.0001. Two-tailed Student's *t*-test, mean ± SEM.

As aforementioned, deletion of *Prmt1* caused eminent testicular atrophy and the resultant male infertility. To decipher at what stage the germ cells were impacted in the *Prmt1-null* testis during the first wave of spermatogenesis, we examined the histology of the testes from Prmt1-sKO mice by hematoxylin and eosin (H&E) staining. We collected the testes at four time points, which correspond to the occurrence of representative germ cell types in testis, including P7 (spermatogonia), P14/17 (early and late spermatocytes), and P21 (spermatids). We found that while the cellular composition and morphology resembled each other between WT and Prmt1-sKO testes at P7, the spermatocytes at P14/17 exhibited aberrant nuclei, reminiscent of cell dying (Figure 5A). At P21, a large number of haploid spermatids present in WT testicular tubules were not seen in the Prmt1-sKO tubules, and the majority of spermatocytes were largely depleted. Detailed counting of the germ cells at each time point indicated that the germline development was arrested at the spermatogonial stage in the Prmt1-sKO testes (Figure 5B). To further verify the findings from the H&E staining, we carried out the immunofluorescence staining on testicular cryosections with two antibodies against Plzf (spermatogonial stem and progenitor cell population) and Ki67 (proliferating spermatogonial cells) respectively. In agreement with the H&E results, at P7, the average numbers of the spermatogonial sub-populations represented by either Plzf or Ki67 staining were comparable in WT and Prmt1-sKO testes (Supplementary Figure S4A–C). However, at P21, the average number of Ki67-positive cells was significantly reduced, in contrast to the similar numbers of Plzf-positive spermatogonial cell population (Supplementary Figure S4D–F). The Ki67 marker has been generally regarded as being representative of the differentiated spermatogonial cell population in the mouse testis. Since the c-Kit marker for differentiated spermatogonia was not effective for immunofluorescence staining, we thus conducted immunoblot to compare the expression levels of c-Kit upon *Prmt1* deletion. We discovered that the levels of c-Kit protein were evidently decreased in the Prmt1-sKO testes at both P8 and P17 days, suggesting that Prmt1 depletion resulted in deficient differentiation of spermatogonial cells (Supplementary Figure S4G). The depleted germ cells likely underwent cell apoptosis, since the TUNEL assay revealed a vast number of positively stained germ cells in the Prmt1-sKO testes at P21(Supplementary Figure S4H-I). This evidence altogether led us to conclude that Prmt1 is required for spermatogonial differentiation and spermatocyte development.

Given that we have previously shown that Stra8-Cre is not proficient in excision of two *loxp* alleles in spermatogonial population (34), it is thus unclear whether Prmt1 is essential for the undifferentiated spermatogonia, i.e. spermatogonial stem and progenitor cells, especially when considering the highest levels of Prmt1 protein detectable in the spermatogonia as described before (Figure 1). To explore this postulation, we next exploited both Tam-inducible Prmt1-uKO and Prmt1-dKO lines and performed the tamoxifen induction during the first wave of spermatogenesis. Due to the lethality of the Prmt1-uKO mice following tamoxifen injection, the testes were allowed for 8 days in Prmt1-uKO mice and 24 days in Prmt1-dKO prior to collection for H&E analyses after three-day tamoxifen injection (Figure 5C, D). This unveiled that both undifferentiated and differentiated spermatogonial cell populations were substantially eliminated from the seminiferous tubules (Figure 5E, F). We further executed co-staining by Sox9 (Sertoli-cell-specific marker) in conjugation with Plzf or Pcna (proliferating spermatogonia) on the testicular cryosections, and uncovered that nearly the whole spermatogonial cell populations were essentially eradicated from the tubules (Figure 5G–J). This evidence shows that Prmt1 is pivotal to orchestrate spermatogonial proliferation and differentiation during the first wave of spermatogenesis.

The maintenance of male fertility in the adult mammalian testis depends on the orderly cycling of spermatogonial cells between self-proliferation and differentiation prior to meiotic division. To interrogate whether Prmt1 is critical for the maintenance of spermatogonia in the adult testis, we took advantage of the Prmt1-dKO line by administering the tamoxifen into the 8-week-old adult mice (Supplementary Figure S4J), and implemented whole-mount immunofluorescence staining with Gfrα1 antibody. In the WT testes, Gfrα1 typically marks undifferentiated spermatogonial population, which is characterized by the As, Apr, and Aal4 spermatogonial cells seen in whole-mount sections. We observed the elongated Pseudopod which is usually indicative of the active stem cell population in the WT testes. By comparison, the number of the Gfrα1-positive spermatogonia and the length of elongated Pseudopod were evidently reduced in the Prmt1-dKO testes at 7-day post-Tam treatment (Supplementary Figure S4K, L). An exhaustive time-course tracking of Tam-treated testes by co-staining with c-Kit and Gfrα1 revealed that the whole spermatogonial pool was essentially eradicated (Supplementary Figure S4M–P), verifying the necessity of Prmt1 in maintaining the homeostasis of spermatogonia in adult testes. Altogether, this evidence unraveled that Prmt1 is a core player required to establish and maintain the developmental trajectory of spermatogonial cells in the testis.

### Prmt1 establishes the high-fidelity RNA transcriptome in spermatogonia

**Figure 6.**
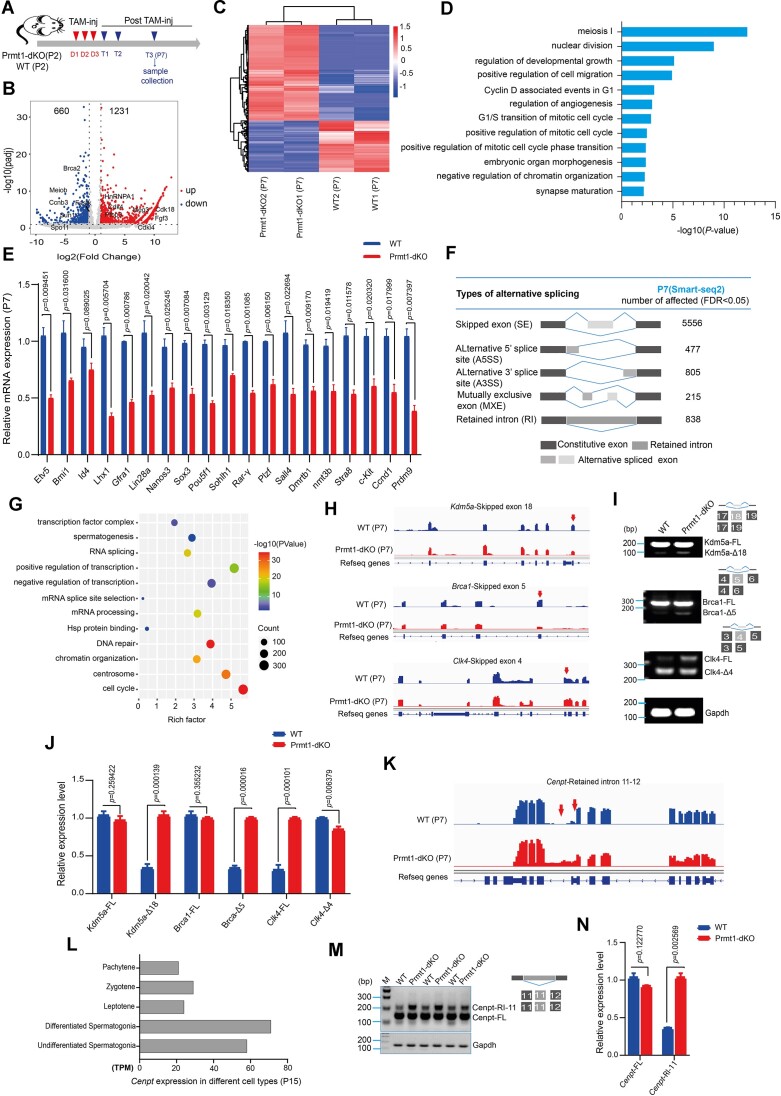
Prmt1 sculpts the transcriptomic identity in spermatogonia. (**A**) The scheme for tamoxifen injection and tissue collection. (**B**) Volcano plot illustrating the dys-regulated genes in the Prmt1-dKO spermatogonia purified by FACS from mice as indicated in ‘A’ using Smart-seq2 approach. (**C**) A heatmap illustrating the dys-regulated genes in Prmt1-dKO spermatogonia compared with WT in biological duplicates. Fold change >2.0, and *P* < 0.05 (upregulated in red; down-regulated in blue). (**D**) GO (gene ontology) terms of differentially expressed genes (DEGs) in FACS sorted, Prmt1-dKO spermatogonia. (**E**) Relative mRNA levels for genes involved in development of spermatogonia from Prmt1-dKO and WT mice (*n* = 3). Two-tailed Student's *t*-test, the data are displayed as the mean ± SEM. (**F**) Statistical calculation of alternative splicing events identified in sorted, Prmt1-dKO spermatogonia via Smart-seq2. (**G**) GO analysis of alternatively spliced genes that are significantly affected in sorted, Prmt1-dKO spermatogonia. (**H**) A snapshot of IGV browser showing mRNA expression levels for Kdm5a, Brca1, and Clk4 in spermatogonia from WT and Prmt1-dKO mice; The red arrow indicates the splicing site. (**I**) RT-PCR verification for Kdm5a, Brca1, and Clk4 genes showing AS pattern of exon skipping (Exon 18, 5 and 4), respectively, in Prmt1-dKO testes; Gapdh was used as an internal control. (**J**) Quantification of the expression for transcripts with skipped exon. FL, full length isoform, Δ represents skipped exon; (*n* = 3). Two-tailed Student's *t*-test, the data are displayed as the mean ± SEM. (**K**) A snapshot of IGV browser showing Cenpt mRNA expression. The red arrow indicates the splicing site. (**L**) mRNA expression levels of Cenpt deduced from single-cell dataset in P15 mouse testis, transcript per million (TPM) (68). (**M**) RT-PCR analysis of Cenpt mRNA expression showing AS pattern of intron 11 retention in Prmt1-dKO testis. (**N**) Quantification of the expression for transcripts with retained intron in ‘M’ (*n* = 3). Two-tailed Student's *t*-test, the data are displayed as the mean ± SEM.

To interrogate how Prmt1 loss impacts the spermatogonia at the transcriptomic level, we next conducted RNA sequencing using either whole mouse testis or purified mouse spermatogonia. The samples were collected at P7 following three consecutive doses of Tam injection (Figure 6A and Supplementary Figure S5A). The spermatogonial population was sorted through Fluorescence-activated Cell Sorting (FACS) by utilizing our *in-house* generated Ddx4-CreERT2 KI mouse model, which carries an EGFP tag driven by the Ddx4 promoter. Conventional bulk RNA-seq using whole mouse testis at P7 identified a large number of dys-regulated genes (1365 up-regulated vs 293 down-regulated) in the Prmt1 KO testes upon Tam treatment (cutoff: fold change ≥ 2, adj-*P*-value < 0.05) (Supplementary Figure S5B, C). As shown above, loss of Prmt1 led to the spermatogonial loss, likely causing a disproportional population of spermatogonia between whole WT and KO testes. Hence, to capture the accurate transcriptomic change, we further performed the mini-Smart-seq2 analysis, which was specifically optimized for low-input cell samples (∼ranging from 10 000–100 000), using FACS-sorted, EGFP-positive spermatogonial cells from P7 testes. Consistently, this revealed 1231 upregulated along with 660 downregulated genes (Figure 6B, C) in Prmt1-dKO spermatogonia (cutoff: fold change ≥ 2, adj-*P*-value < 0.05). GO enrichment analysis unveiled that those dys-regulated genes are predominantly involved in cell-cycle and meiotic regulation (Figure 6D). Notoriously, in support of previous phenotypic defects seen in *Prmt1-null* spermatogonia, a handful of genes involved in spermatogonial self-renewal (Id4, Plzf, Bmi1, etc.) and differentiation (Ccnd1, C-kit, Sohlh-1, etc.) were markedly reduced in Prmt1-dKO spermatogonia as validated by qPCR (Figure 6E), suggesting Prmt1 is crucial to establish and maintain the transcriptomic integrity of spermatogonia.

PRMT enzymes are known to impact alternative splicing through methylating the splicing factors, e.g. SmB and hnRNP members (35–38). We thereby analyzed alternative splicing (AS) events, which were divided into five categories—skipped exon (SE), Alternative 5′ splice site (A5SS), Alternative 3′ splice site (A3SS), mutually exclusive exon (MXE) and retained intron (RI). Among them, the SE dominates the alternative splicing events as detected by both whole bulk RNA-seq and mini-Smart-seq analyses (Figure 6F and Supplementary Figure S5D, E). GO examination unraveled the AS genes are enriched in cell-cycle regulation and mRNA processing (Figure 6G). A visual inspection of the integrative genomic viewer (IGV) for AS genes with SE, such as Kdm5a, Brca1, Clk4, Srsf9, Map7, Ccna1, Srsf7 and Lsm5, was further corroborated by the semi-quantitative PCR validation, showing that those exons were aberrantly skipped or retained in the *Prmt1-null* spermatogonia (Figure 6H–J and Supplementary Figure S5F–N). Moreover, the CENP gene family, including CENP-C, CENP-H, CENP-M, CENP-N, CENP-U and CENP-T, assembles the CENP-A NAC complex, which safeguards chromosomal alignment and segregation (39). Those members were all dys-regulated upon Prmt1 loss as revealed by Smart-seq2 examination. Among them, Cenpt is prominently enriched in spermatogonial population, and both IGV browser snapshot and qPCR inspection verified that the intron 11 is significantly retained in the *Prmt1-null* spermatogonia (Figure 6K–N). Together, this evidence indicates that Prmt1 maintains the transcriptomic high-fidelity essential for spermatogonial development.

### Global profiling identified the genomic distribution of histone arginine methyl marks and a direct competition between H4R3me2s and H4R3me2a at promoters in regulating gene expression

**Figure 7.**
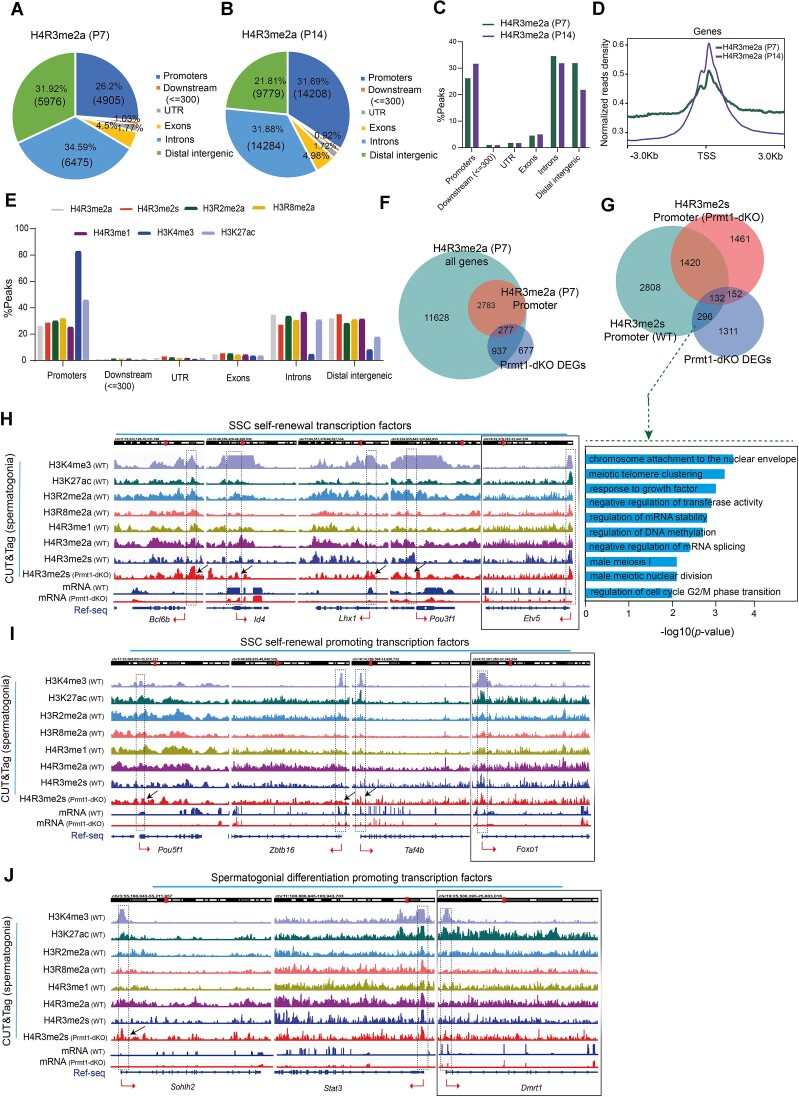
Global profiling identified the genomic landscape of histone arginine methyl marks and a direct competition between H4R3me2s and H4R3me2a at promoters in regulating gene expression. (**A**, **B**) Genomic distribution of H4R3me2a marks by CUT&Tag profiling in the WT mouse testes at P7 and P14. (**C**) Distribution of genomic loci for H4R3me2a in the WT mouse testis at P7 and P14. (**D**) Enrichment of H4R3me2a at TSS region in the WT mouse testis at P7 and P14. (**E**) Comparative analysis of genomic distribution for five histone methylarginine marks, plus active marks for promoter (H3K4me3), and enhancer (H3K27ac) in WT testis at P7. (**F**) Gene overlapping among H4R3me2a-P7 all genes, H4R3me2a enriched at promoters, and Prmt1-dKO DEGs. (**G**) Overlapping genes for H4R3me2s enriched at promoters (WT) and H4R3me2s enriched at promoters (Prmt1-dKO) and Prmt1-dKO DEGs; GO terms for 296 genes that are only shared by WT H4R3me2s and Prmt1-dKO DEGs. (**H–J**) IGV browser snapshot showing the peak distribution for histone methylarginine marks and mRNA gene expression for SSC self-renewal transcription factors, SSC self-renewal promoting transcription factors and spermatogonial differentiation promoting transcription factors.

Prmt1 deposits the asymmetrical H4R3 methylation (H4R3me2a) known as an active histone mark for gene expression. To explore the mechanism by which Prmt1 loss caused the transcriptomic dys-regulation, we attempted to execute the highly sensitive and efficient CUT&Tag profiling recently developed in Henikoff's Lab (27). As a fact, the conventional ChIP-seq assay rarely succeeded in identifying the genome-wide distribution of histone arginine methyl marks in the past decades (as reviewed (40)). To enrich the spermatogonia in P7 testes, we removed the Sertoli cells by filtering the single-cell suspension through 40 μm strainer, and determined that the ratio of germ cells to somatic cells in the filtrate exceeded 95% (Supplementary Figure S6). Next, we selected ChIP-qPCR validated antibodies against the histone methylarginine marks with compensatory increases in the Prmt1-dKO testes, including H4R3me2a for Prmt1, H3R2me2a for Prmt6, H4R3me2s for Prmt5, H3R8me2a for Prmt2 and H4R3me1 for Prmt7. Meanwhile, both H3K4me3 and H3K27ac were included for comparison, which specifically delineates promoter and enhancer elements respectively. This high-throughput profiling revealed > 14000 peaks on average for each methyl mark with high confidence (Cutoff: *P* < 0.005) (Supplementary Figure S7). Strikingly, all five histone arginine methyl marks (H4R3me2a, H3R2me2a, H4R3me2s, H3R8me2a, H4R3me1) are highly enriched in the TSS region, albeit with much lower peak densities, compared with the H3K4me3 peak at the TSS region (Figure 7A-C, Supplementary Figures S7A–N and S8A–I). Moreover, there is a high overlapping of the TSS peaks among the five methylarginine marks, H3K4me3 and H3K27ac (Supplementary Figure S8C). An overlapping between the H4R3me2a-enriched peaks and DEGs in *Prmt1-null* spermatogonia unveiled that a large number of genes, in particular, among which a total of 277 genes, are regulated by the Prmt1-deposited H4R3me2a (Figure 7F). This signified that PRMT members are capable of directly tuning gene expression by depositing histone methylarginine marks at gene promoters.

Prior studies have documented that Prmt1-deposited H4R3me2a most often functions as an activator, while Prmt5-catalyzed H4R3me2s predominantly functions as a repressor (41,42). Since both marks occur at the same arginine residue in histone 4, it is thus tempting to postulate that the intricate competition between Prmt1 and Prmt5 likely determines the ultimate outcome of gene expression at specific genomic loci. We thus next assessed how H4R3 methylation is impacted in the absence of Prmt1. Surprisingly, we found that the H4R3me2s intensity was reduced to one-half in the Prmt1-dKO testes as compared to that in WT testis at P7 (Supplementary Figure S8I–L). Among a total of 3165 promoter peaks for H4R3me2s identified in the Prmt1-dKO testes, approximately a half (1613) were newly synthesized, and this might directly finetune the increased expression levels for Prmt2/5/6 in Prmt1-deficient germ cells (Supplementary Figure S8J–L). Further overlapping with H4R3me2a-enriched peaks at TSS region showed that a total of 330 H4R3me2s peaks competed for the endogenous H4R3 loci (Supplementary Figure S8J). In addition, the overlapping between H4R3me2s-enriched TSS peaks and dys-regulated genes uncovered that a total of 296 affected genes lost the H4R3me2s mark while 152 genes gained new H4R3me2s decoration (Figure 7G). This evidence suggests that the endogenous competition between H4R3me2s and H4R3me2a likely accounted for the aberrant transcriptomic regulation in the *Prmt1-null* spermatogonia. We therefore next visually inspected the IGV browser for a panel of transcription factors essential for spermatogonial development, which exhibited down-regulated expression upon loss of Prmt1. They can be divided into three categories - SSC self-renewal, SSC self-renewal promoting, and spermatogonial differentiation promoting transcription factors (Figure 7H-J). The IGV tracks comprise a total of five methylarginine marks as well as two well-known active marks (H3K4me3 and H3K27ac) as positive controls and for promoter identification. This exhaustive examination revealed that, in support of the down-regulated mRNA levels, the repressive H4R3me2s peak intensities for three SSC self-renewal transcription factors (Bcl6b, Id4, Lhx1 and Pou3f1) were significantly elevated in their TSS regions in the Prmt1-dKO testes at P7 (Figure 7H). By comparison, there is no difference for H4R3me2s peak enrichment in the TSS region for Etv5, which showed comparable mRNA expression levels between WT and Prmt1-dKO testes (Figure 7H). Further visual inspections for three SSC self-renewal promoting transcription factors (Pou5f1, Zbtb16 and Taf4b) (Figure 7I) and spermatogonial differentiation promoting transcription factor (Sohlh2) (Figure 7J) are also supportive of the notion that re-occupancy of H4R3me2s upon *Prmt1* loss at the promoters, at least in part, accounted for the down-regulated mRNA levels for genes that are critical to maintain spermatogonial homeostasis (Figure 7H–J). Together, this evidence suggests that Prmt1-deposited H4R3me2a intrinsically competes with H4R3me2s to establish transcriptomic identity that coordinates spermatogonial self-renewal and differentiation.

### Evidence that H4R3 methylation governs alternative splicing through directly regulating expression of splicing-related factors as well as shaping of local chromatin signature

**Figure 8.**
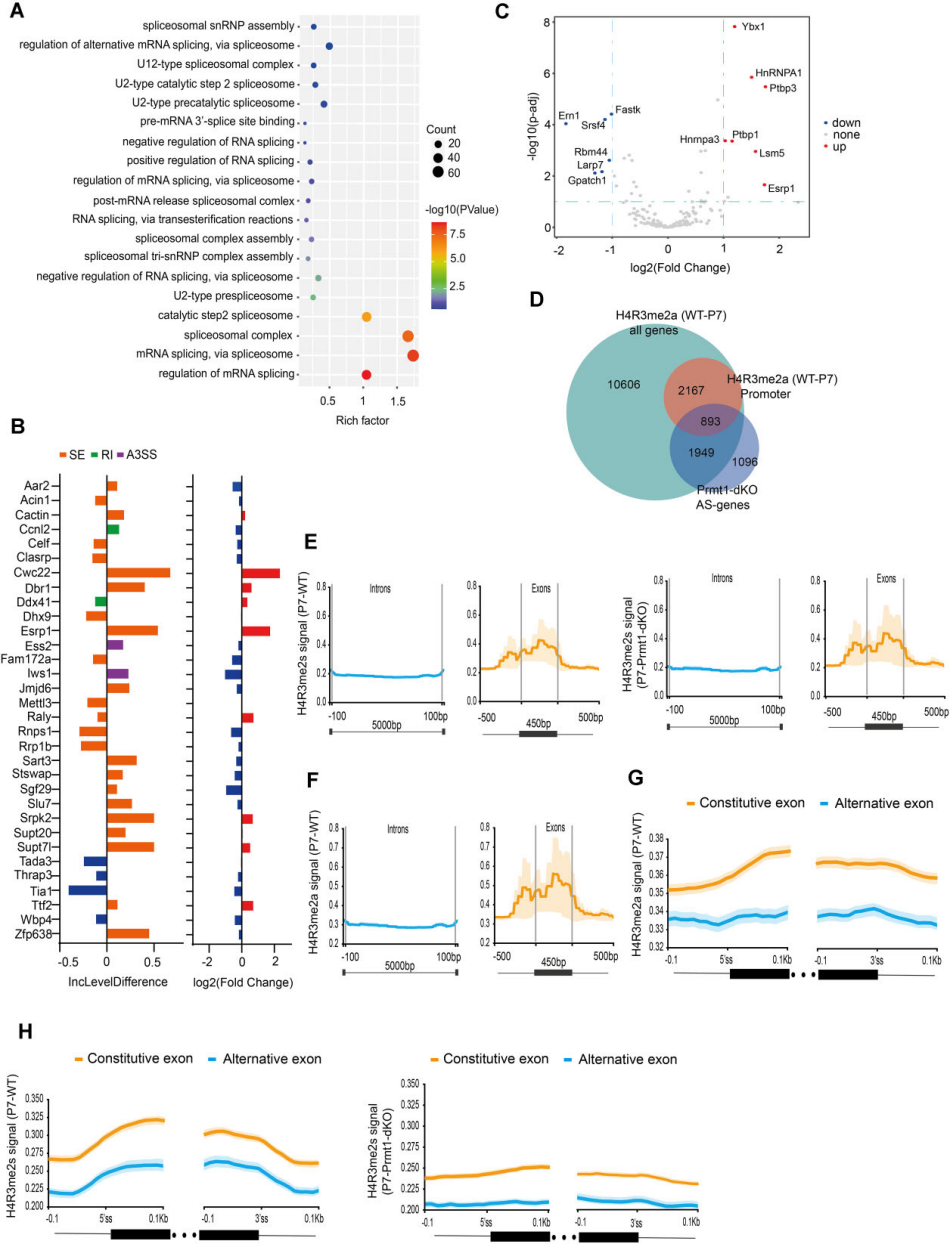
H4R3 methylation governs alternative splicing through directly regulating expression of splicing-related factors as well as indirect shaping of local chromatin signature. (**A**) GO enrichment for RNA splicing-related genes identified by Smart-seq2 in the Prmt1-dKO spermatogonia. (**B**) Differential expression and inclusion level difference for three types of splicing events in RNA splicing-related genes. >0 indicates a difference in the degree of inclusion in Prmt1-dKO and <0 reflects inclusion in WT. (**C**) Volcano plot depicting the dys-regulated expression of RNA splicing-related genes. (**D**) Gene overlapping among H4R3me2a-enriched genes, H4R3me2a enriched at promoters, and Prmt1-dKO AS genes. (**E**, **F**) Comparison of the averaged peak intensities for H4R3me2s and H4R3me2a enrichment between introns and exons deduced from CUT&Tag. The average lengths for exons are ∼450 bp while intron lengths averaging ∼5 kb. (**G**, **H**) **C**omparison of the averaged peak enrichment intensities for H4R3me2s and H4R3me2a across the 100 bp flanking regions for alternative and constitutive exons.

Since we have attained a global landscape of histone methylarginine mark distribution, we next evaluated how the histone marks are linked to the aberrant alternative splicing as seen in *Prmt1-null* spermatogonia. An examination of Smart-seq2 data identified that >5000 transcripts for a total of 3938 genes were aberrantly spliced in mouse spermatogonia (Figure 6F). Among them, GO enrichment showed that a group of defectively spliced transcripts are closely linked to splicing machinery assembly and function (Figure 8A), and displayed skipped exon (SE), retained intron (RI) and alternative 3′ splice site (A3SS) (Figure 8B and Supplementary Figure S9A–C). Some of them, such as Srsf4, HnRNPA1 and Ptbp1, were significantly dys-regulated in the *Prmt1*-*null* spermatogonia (Figure 8C). Likewise, Bud31- and Ddx5-mediated alternative splicing in SSCs governs the differentiation and self-renewal of mammalian male germ cells (43,44). This evidence suggests that Prmt1 likely coordinates alternative splicing indirectly by regulating expression of splicing factors. Since H4R3 methyl marks are highly enriched in the promoter region of protein-coding genes as discovered by CUT&Tag profiling, we next asked whether there is a direct regulation of the AS-regulating transcripts by H4R3 methylation. The overlapping with the H4R3me2a-enriched peaks at promoters identified a total of 893 defectively splicing genes in the Prmt1-dKO spermatogonia, which are presumably under the direct transcriptional control of Prmt1-catalyzed H4R3me2a modification. H4R3me2a is enriched on promoter and gene body that help in normal transcription initiation. We argued that these modifications influence alternative splicing because transcription is dependent on arginine histone PTM and those transcribed exons are spliced further to mature transcript as we identified 1949 genes that were overlapped with defectively spliced genes (Figure 8D). This evidence suggests that Prmt1 is capable of directly modulating the alternative splicing, at least in part, by H4R3me2a deposition at promoters for splicing-related factors.

Surprisingly, distinct from the known peak enrichment at promoters for the active H3K4me3 (>80%) and H3K27ac (>40%) marks (Supplementary Figure S8G-H), the global CUT&Tag profiling uncovered prominent proportions of peaks enriched at the exons (∼4%) and introns (∼30%) for the five methylarginine marks (Figure 7A–E and Supplementary Figure S8D–I). This led us to speculate that these methyl marks likely execute other regulatory functions, in addition to the fraction of peaks distributed at promoters in driving gene expression. Indeed, emerging evidences have shown that the non-random distribution of H3K36me3 occupies more frequently in exonic than in intronic regions in the protein-coding gene bodies, and this is intimately linked to the characteristic alternative splicing pattern in a context-specific manner (45). To test this hypothesis, we first assessed how the various types of histone marks correlate with each other for the SE and RI splicing events in mouse spermatogonia. Pearson correlation analyses unveiled that there is closer relationship of the peaks among H4R3me2a, H3R2me2a, H4R3me1 and H3R8me2a (Pearson correlation coefficient > 0.69) (Supplementary Figure S9D, E). By comparison, there is a less correlation for H4R3me2s (Pearson correlation coefficient ≈ 0.4) as compared to other four histone arginine methyl marks (Supplementary Figure S9D, E), suggesting there might exist a counteractive effect between H4R3me2s and H4R3me2a (possibly other methylarginine marks) during RNA transcription. As a control, the H3K4me3 and H3K27ac marks are known as active marks without known functional roles in regulating exon skipping or intron retention, and are thereby not surprising to cluster separately. Next, we extracted the intronic and exonic regions with 500-bp flanking sequences across the exon-intron junctions separately for the aberrantly spliced genes, and calculated the averaged read tag densities for different histone modification marks. This uncovered that, consistent with the reported selective distribution of H3K36me3 mark involved in splicing (45,46), the peaks for H4R3me2a, H4R3me2s and H3R2me2a are highly enriched in the exonic regions, but not in the intronic regions (Figure 8E-F and Supplementary Figure S9F-H). On the other hand, for the alternatively spliced genes, we isolated both the constitutive and alternative exon regions with flanking 100-bp intronic sequence, and calculated the read density enrichment across the exon-intron junctions. In support of previous findings, there exists an apparent enrichment of H4R3me2s and H4R3me2a peaks for both the 5′- and 3′- ends across exon-intron junctions, but not for the H3K4me3 and H3K27ac peaks (Figure 8G, H and Supplementary Figure S9IK). In the *Prmt1-null* spermatogonia, the enrichment trend for H4R3me2s peaks completely disappeared (Figure 8H), indictive of a reciprocal interplay between H4R3me2s and H4R3me2a in regulating alternative splicing of the exons/introns in spermatogonia. In summary, these data provide evidence that histone methylarginine-directed chromatin signature, consisting of H4R3me2s and H4R3me2a, harbors profound impact on alternative splicing in spermatogonial development.

## DISCUSSION

Maintenance of male fertility hinges on continuous spermatogenesis, which is established and sustained through the timely self-renewal and co-ordinated differentiation programming in the highly heterogeneous population of spermatogonial cells in the mammalian testis. Herein, we identified a core epigenetic factor, namely Prmt1, which is required to coordinate spermatogonial development through (i) governing gene expression for germline transcription factors associated with spermatogonial self-renewal and differentiation; (ii) modulating the expression of splicing-related factors by depositing H4R3me2a at gene promoters; (iii) fine-tuning the alternative splicing through delineation of H4R3me2a mark at exonic regions.

### Genome-wide profiling of histone methylarginine marks by CUT&Tag identified the permissive but low-abundance distribution at promoters

To gain a deep understanding of epigenetic regulation at the molecular level, it is of paramount importance to attain a thorough landscape of genome-wide distribution loci for a specific histone PTM. As described above, arginine methylation is one of the widespread histone PTMs occurring in mammalian cells, yet good datasets generated by ChIP-seq approach are by far rarely available. An exhaustive scrutiny of the published papers discovered that the global distribution of most PRMTs and their deposited histone arginine methyl marks is verified through conventional ChIP-PCR or ChIP-qPCR (40). Only until recently, the genomic distribution loci for H3R17me2a (47), H4R3me2s (48) and H3R2me2a (49,50), which are catalyzed by Prmt4, 5 and 6, respectively, have been published using ChIP-seq. In this study, after verifying the validity of the antibodies for immunoprecipitation, we chose to utilize the recently developed CUT&Tag approach to profile the genomic loci distribution in the developing mouse testis. We found that all five histone arginine methyl marks (H4R3me2a, H3R2me2a, H4R3me2s, H3R8me2a, H4R3me1) are in general ubiquitously enriched at promoter regions. However, their enrichment intensities are much lower than that of H3K4me3 in actively transcribed genes (Figure 7 and Supplementary Figure S8). Moreover, their enrichment levels increase substantially from P7 (spermatogonia) to P14 (meiotic spermatocytes) at promoters during postnatal testicular development (Supplementary Figure S8), suggesting that the germline development is subject to dynamic expression regulation during mitosis-to-meiosis transition.

In addition, prior studies have reported that CpG islands (CGIs) are frequently present in the promoter regions, and are often differentially methylated in a cell context-specific pattern (51–55). We therefore extracted and divided the promoter peaks into two proportions: CGI peaks and non-CGI peaks (Supplementary Figure S7). Overall, we discovered that CGI promoters are more likely subject to modulation by histone arginine methylation as compared to the non-CGI promoters in the developing testis from P7 to P14, presumably indicating a synergistic role of arginine methylation along with DNA methylation directly in controlling germline gene expression.

We reasoned that the success of CUT&Tag approach that outcompetes conventional ChIP-seq in profiling the histone arginine methyl marks could be attributed to several aspects. First, the whole steps of target chromatin capture and DNA fragmentation for CUT&Tag were executed on live cells without any fixation step. It is generally accepted that the typical fixative used in conventional ChIP-seq, e.g. formaldehyde, can mask the target antigen causing failure of antibody recognition (56). This assumption is manifested by a more recent study, which unambiguously demonstrated that H3K36me3 is also highly enriched in gene promoters, in addition to the gene body enrichment, by utilizing both CUT&Tag and native ChIP-seq (N-ChIP-seq) methods (57). In contrast, the crosslinking ChIP-seq (X-ChIP-seq) seldomly identified the H3K36me3 in the promoters but exclusively detected the H3K36me3 in the gene body regions, as often reported by previous studies (58,59). Second, although histone arginine methylation is permissively distributed across the genome, we and others found that the methylation levels in histones are generally very low as compared with those of histone lysine methylation (Supplementary Figures S7 and S8). In a typical ChIP-seq library preparation pipeline, the immunoprecipitated DNA fragment is directly subject to end repair and adaptor ligation for library preparation (60). There is a lack of further target-specific DNA enrichment step that can distinguish them from the background DNA noise as carried over by the non-specific binding of both beads and antibodies. In comparison, CUT&Tag executes the target DNA fragmentation and adapter ligation *in situ*, without further introduction of non-target genomic DNA fragments into the library, thereby yielding extremely low background signals. As such, it is advisable to exploit CUT&Tag, rather than conventional fixative/sonication-based ChIP-seq approach, to profile low-abundance histone PTMs, such as arginine methyl marks.

### Arginine methyl marks cross-talk and are required to coordinate spermatogonial development

Prior studies showed that Prmt5 exhibits a dynamic expression pattern in the germline. It is abundant in the cytoplasm of PGC at embryonic day 7.5 (E7.5), followed by shuttling to the PGC nuclei prior to the sex differentiation at E12.5(14). In the developing testis, it is highly detectable in the nuclei of pro-spermatogonia, and gradually translocates to the cytoplasm in the differentiating spermatogonia and early stage of spermatocytes (61). The nuclear Prmt5 is important to restrict the retrotransposon activity by depositing the repressive H4R3me2s mark in PGCs and possibly in postnatal mouse germline cells. Compared with somatic organs, we found that the primary Type I PRMT enzyme -Prmt1- displays higher protein expression levels in the nuclei of germline in developing testis (Figure 1), in particular in spermatogonia and early stages of meiotic spermatocytes. In line with its expression pattern, we generated three germline-specific and Tam-inducible KO mouse models, and provided genetic evidence showing that inactivation of Prmt1 remarkably impeded the timely differentiation and self-renewal in the spermatogonial cells, implicating a central role of nuclear Prmt1 in establishment and maintenance of spermatogonial identity in testis (Figures 2, 5 and Supplementary Figure S4).

With the pan-methylarginine antibodies against MMA, ADMA and SDMA marks, compelling evidence showed that loss of Prmt1 activity induced a remarkable increase in MMA and SDMA levels of substrate methylation, but not in ADMA levels, in somatic MEF cells (32). This evidence suggests a direct cross-talk between Prmt1-directed asymmetric arginine methylation and Prmt5/7-mediated SDMA/MMA deposition. In other words, there exists a substrate competition among three types of arginine methylation. However, in the spermatogonia of the developing testis, we discovered that Prmt1 loss resulted in an eminent increase in both the ADMA and MMA levels, along with a slight increase in SDMA levels, which resulted from the compensatory expression of Prmt2/5/6/7 (Figure 4). By comparison, only elevated expression of Prmt5 was observed in spleen tissue. This signifies that those different members from the same Type I PRMT family, e.g. Prmt1, Prmt2 and Prmt6, as evidenced by both mRNA and protein verification, compete for each other in the germline, but not in the somatic tissues, such as spleen. On the other hand, our genome-wide CUT&Tag data showed that, in the *Prmt1-null* spermatogonia, the peak intensities of H4R3me2s were generally decreased at the promotor loci of Prmt2/5/6 genes. Since H4R3me2s is known as a repressive mark, we speculate that the decreased H4R3me2s occupancy levels might contribute to the elevated expression levels for Prmt2/5/6 in germ cells.

Interestingly, Prmt2 has been traditionally considered as an enzyme-dead arginine methyltransferase owing to its extremely weak enzymatic activity seen *in vitro* (62). Nonetheless, recent studies have unraveled its catalytic activity on asymmetric dimethylation of histone H3R8 (H3R8me2a), which is associated with the oncogenic activation of glioblastoma (63). Our genome-wide profiling by CUT&Tag also revealed that Prmt2-deposited H3R8me2a is capable of modulating gene expression synergistically with other arginine marks, since all five histone arginine methyl marks were abundantly enriched at gene promoters.

### Arginine methyl signaling is important but play distinct functions during spermatogonial development and meiotic divisions

We have previously reported that genetic ablation of Prmt4/Carm1, which is present in the cytoplasm of both spermatogonia and spermatocytes but shuttles to the nuclei of haploid spermatids, severely impaired the elongation of haploid spermatids without apparent defects seen in meiotic spermatocytes (13). In this study, we found Prmt1 is localized abundantly in the nuclei of spermatogonia, but weakly in the nuclei of spermatocytes (Figure 1G and Supplementary Figure S1C). In both the Prmt1-sKO and Prmt1-dKO males treated by Tam, we observed aberrant meiotic spermatocytes and loss of early stages of spermatocytes (Figure 5 and Supplementary Figure S4), implying that Prmt1 is necessary for the development of both spermatogonia and meiotic cell-cycle progression in the testis.

By the CUT&Tag profiling, we consistently observed the increased enrichment of all five methyl arginine marks at gene promoters in spermatocytes (P14) as compared to spermatogonia (P7), which is concomitant with the simultaneous increase in H3K27ac enrichment, whereas there is no difference in H3K4me3 enrichment at promoters (Figure 7 and Supplementary Figure S8). This presumably suggests that Prmt1 might play a significant function in meiotic division, which is distinguished from that in spermatogonia.

### Evidence supporting that histone arginine methyl marks constitute a chromatin signature that is linked to transcriptional alternative splicing

It is known that the pre-mRNA processing is co-transcriptionally coupled to the gene transcription, and emerging evidence supports that there exists co-transcriptional splicing of alternative exons and introns concomitant with RNA transcription (45,64). Therefore, it is conceivable that a combinatorial chromatin signature will impact the transcriptional outcome, including alternative splicing, in a context-dependent manner. However, often these biological events were studied independently owing to the high complexity of factors involved in the transcriptional processing. Recent evidence has provided clues that relate histone modification marks functionally to alternative splicing (65). For instance, integrative analyses of large-scale RNA-seq and ChIP-seq datasets, in conjugation with machine learning, revealed that the histone marks, such as H3K36me3, H3K79me2 and H3K4me1, are functionally relevant to exclusion/inclusion of exons and introns in a variety of cellular processes (66). Among the known AS-related histone marks, H3K36me3 is the only fairly-studied example involved in numerous cellular events, and its mode of action is relatively clear. For instance, H3K36me3 is recognized by the chromodomain of MORF-related gene on chromosome 15 (Mrg15), which recruits the splicing regulator PTB1 to modulate alternative splicing (67). Among diverse cellular contexts, H3K36me3 is often highly enriched in the exonic regions of the protein-coding genes, along with the promoter regions as recently discovered by the optimized CUT&Tag profiling (57). Strikingly, we also unveiled that all five histone arginine methyl marks are distributed in the exon/intron regions in addition to the promoter enrichment. Exhaustive analyses suggest that the intricate interplay among these histone marks is responsible for the characteristic splicing pattern in the germline essential for male fertility.

## Supplementary Material

gkad769_Supplemental_File

## Data Availability

All the sequencing data for RNA-seq, Smart-seq2 and CUT&Tag are publicly available in the Gene Expression Omnibus database under the Accession Code GSE227857.
