# Supplementary material for: The arginine methyltransferase Prmt1 coordinates the germline arginine methylome essential for spermatogonial homeostasis and male fertility
Source: Nucleic Acids Res. 2023 Sep 22;51(19):10428–50. doi: 10.1093/nar/gkad769 (PMC10602896; doi:10.1093/nar/gkad769)
Supplement: gkad769_Supplemental_File [file gkad769_supplemental_file.pdf]

Supplementary information for

The arginine methyltransferase Prmt1 coordinates the germline arginine methylome essential for spermatogonial homeostasis and male fertility

Muhammad Azhar,[1,2,†] Caoling Xu,[1,2,†] Xue Jiang,[1,2] Wenqing Li,[1,2] Yuzhu Cao,[1,2] Xiaoli Zhu,[1,2] Xuemei Xing,[1] Limin Wu,[1] Jiaqi Zou,[1,2] Lan Meng,[1,2] Yu Cheng,[1,2] Wenjie Han,[2] and Jianqiang Bao[1,2,*]

[1]Department of Obstetrics and Gynecology, Reproductive and Genetic Hospital, The First Affiliated Hospital of USTC, Division of Life Sciences and Medicine, University of Science and Technology of China, Hefei, Anhui, 230001, China

[2]Hefei National Laboratory for Physical Sciences at Microscale, Biomedical Sciences and Health Laboratory of Anhui Province, University of Science and Technology of China (USTC), Anhui, China

[†] The authors wish it to be known that, in their opinion, the first two authors should be regarded as Joint First Authors

[*]Corresponding author

Jianqiang Bao, PhD. Principal Investigator.

Email: jqbao@ustc.edu.cn

Tel: +86 551 63606389

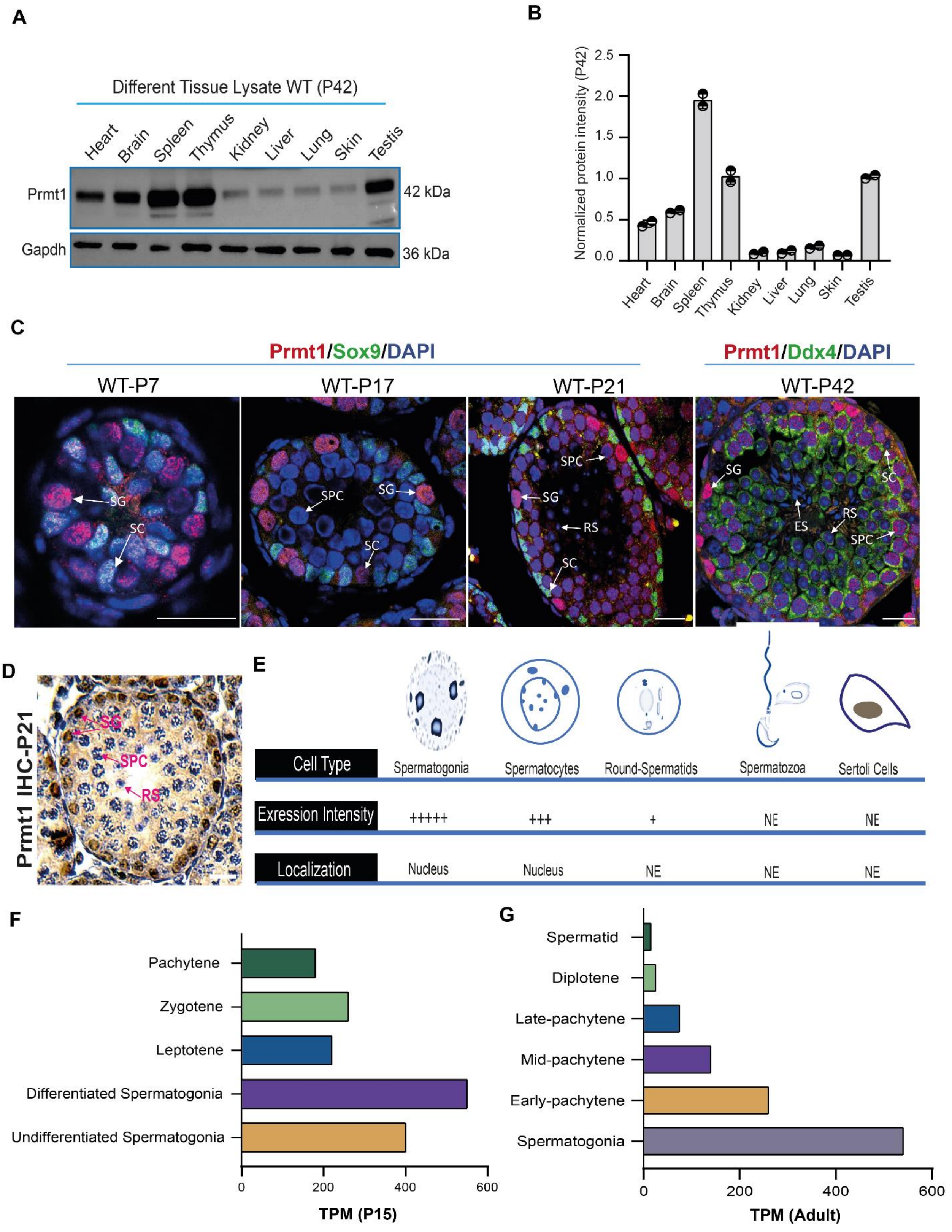
A
Different Tissue Lysate WT (P42)
Heart
Brain
Spleen
Thymus
Kidney
Liver
Lung
Skin
Testis
Prmt1
42 kDa
Gapdh
36 kDa
B
Normalized protein intensity (P42)
2.5
2.0
1.5
1.0
0.5
0.0
Heart
Brain
Spleen
Thymus
Kidney
Liver
Lung
Skin
Testis
C
Prmt1/Sox9/DAPI
Prmt1/Ddx4/DAPI
WT-P7
WT-P17
WT-P21
WT-P42
SG
SC
SPC
RS
ES
D
Prmt1 IHC-P21
SG
SPC
RS
E
Cell Type
Spermatogonia
Spermatocytes
Round-Spermatids
Spermatozoa
Sertoli Cells
Exression Intensity
+++++
+++
+
NE
NE
Localization
Nucleus
Nucleus
NE
NE
NE
F
Pachytene
Zygotene
Leptotene
Differentiated Spermatogonia
Undifferentiated Spermatogonia
0
200
400
600
TPM (P15)
G
Spermatid
Diplotene
Late-pachytene
Mid-pachytene
Early-pachytene
Spermatogonia
0
200
400
600
TPM (Adult)

Supplementary Fig S1. Prmt1 is highly expressed and predominantly localized to the nucleus in spermatogonia but weakly in spermatocytes in mouse testes

(A) Immunoblotting of Prmt1 in WT mouse tissues at P42. Gapdh served as a loading control. (B) Quantitation of normalized protein intensity for Prmt1 in WT mice at P42 from ‘A’ (n = 2). (C) Immunostaining of Prmt1 and Sox9/Ddx4 in testicular sections from WT mice at P7, P17, P21 and P42. SG-spermatogonia, SPC-spermatocyte, SC-Sertoli cell, RS-round spermatids, ES-elongating spermatids. Scale bars, 20 µm. (D) Immunohistochemical staining of Prmt1 in juvenile mouse testes at P21. SG-spermatogonia, SPC-spermatocyte, RS-round spermatids (E) A schematic illustration depicting the expression of Prmt1 at different types of germ cells in testis; “+” represents the intensity of Prmt1 protein levels, NE - no expression. (F) Dynamic Prmt1 mRNA expression pattern in germ cells from P15 juvenile mice inferred from single-cell datasets (1). (G) Dynamic expression of Prmt1 mRNA levels in adult mouse testes inferred from single-cell datasets (1).

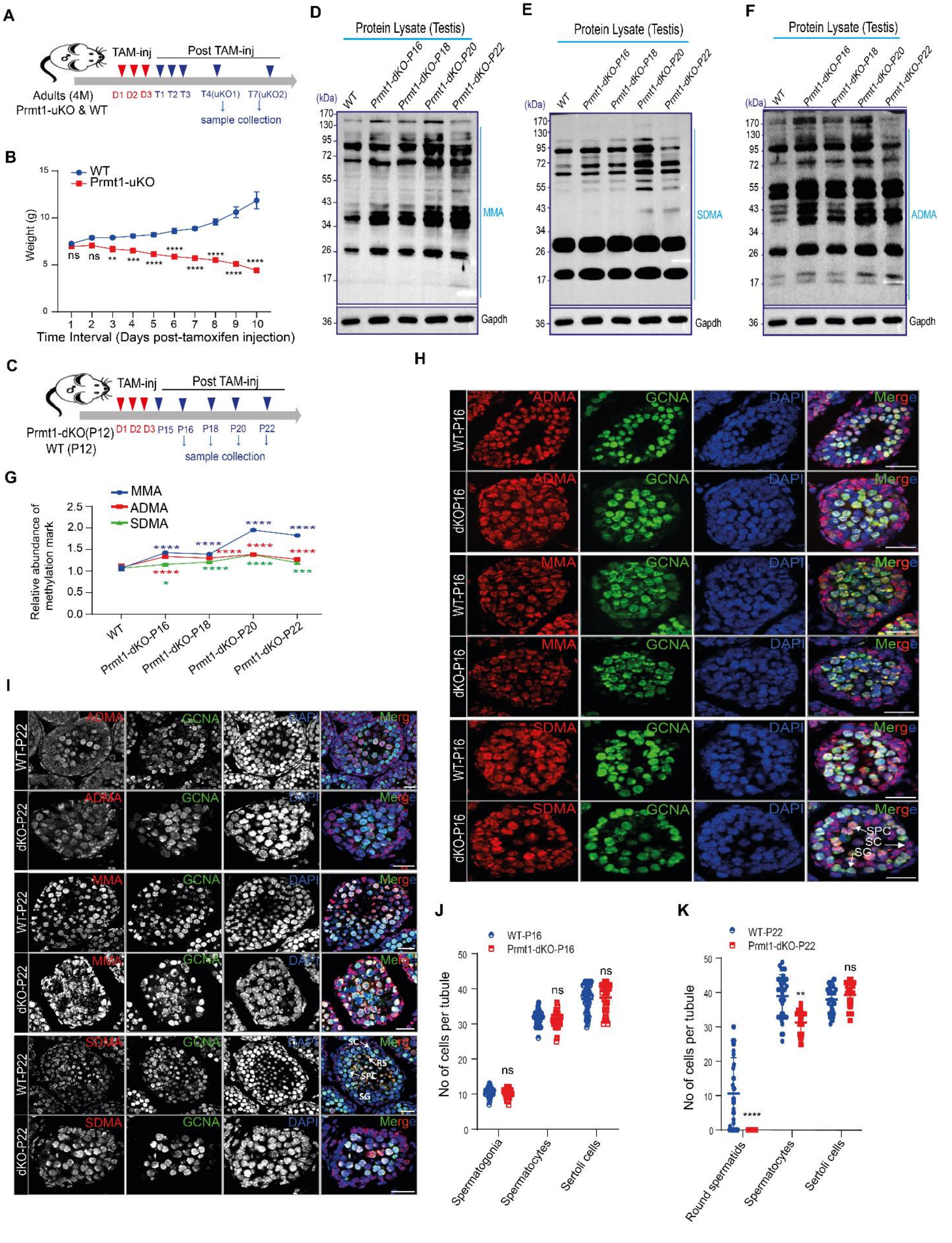

A
TAM-inj
Post TAM-inj
D1 D2 D3 T1 T2 T3 T4(uKO1) T7(uKO2)
Adults (4M)
Prmt1-uKO & WT
sample collection
B
WT
Prmt1-uKO
Weight (g)
Time Interval (Days post-tamoxifen injection)
C
TAM-inj
Post TAM-inj
D1 D2 D3 P15 P16 P18 P20 P22
Prmt1-dKO(P12)
WT (P12)
sample collection
D
Protein Lysate (Testis)
WT Prmt1-dKO-P16 Prmt1-dKO-P18 Prmt1-dKO-P20 Prmt1-dKO-P22
MMA
Gapdh
E
Protein Lysate (Testis)
SDMA
Gapdh
F
Protein Lysate (Testis)
ADMA
Gapdh
G
MMA
ADMA
SDMA
Relative abundance of methylation mark
H
ADMA GCNA DAPI Merge
WT-P16
dKOP16
MMA
dKO-P16
SDMA
SPC
SC
SG
I
WT-P22
dKO-P22
RS
J
WT-P16
Prmt1-dKO-P16
No of cells per tubule
Spermatogonia
Spermatocytes
Sertoli cells
K
WT-P22
Prmt1-dKO-P22
Round spermatids

Supplementary Fig. S2. Prmt1 modulates the substrate arginine methylome in the germline distinct from somatic cells

(A) Tamoxifen injection scheme in the adult mice and tissue collection from Prmt1-uKO mice. D, Day. T, post-tamoxifen injection. (B) Body weights of mice at days following post-tamoxifen injection in Prmt1-uKO mice; *p<0.05, **p< 0.01, ***p< 0.001, and ****p<0.0001; mean±SEM, and n=3. (C) The scheme for tamoxifen injection and tissue collection from Prmt1-dKO mice. (D-F) Immunoblotting on testicular lysate with MMA, SDMA, and ADMA antibodies, respectively in WT and Prmt1-dKO mice at indicated times 'C'. Gapdh as an internal control. (G) Quantification of substrate methylarginine intensities from 'D-F'. *p<0.05, **p<0.01, ***p<0.001, and ****p<0.0001. (H-I) Immunostaining of ADMA, MMA, and SDMA substrates, with co-staining by GCNA in testis sections from WT and Prmt1-dKO mice at indicated times 'C'. Scale bars, 20 µm. (J-K) Quantification of average numbers of germ cells at indicated times 'C' from 'H-I', mean±SEM, and n=3. **p< 0.01 and ****p<0.0001.

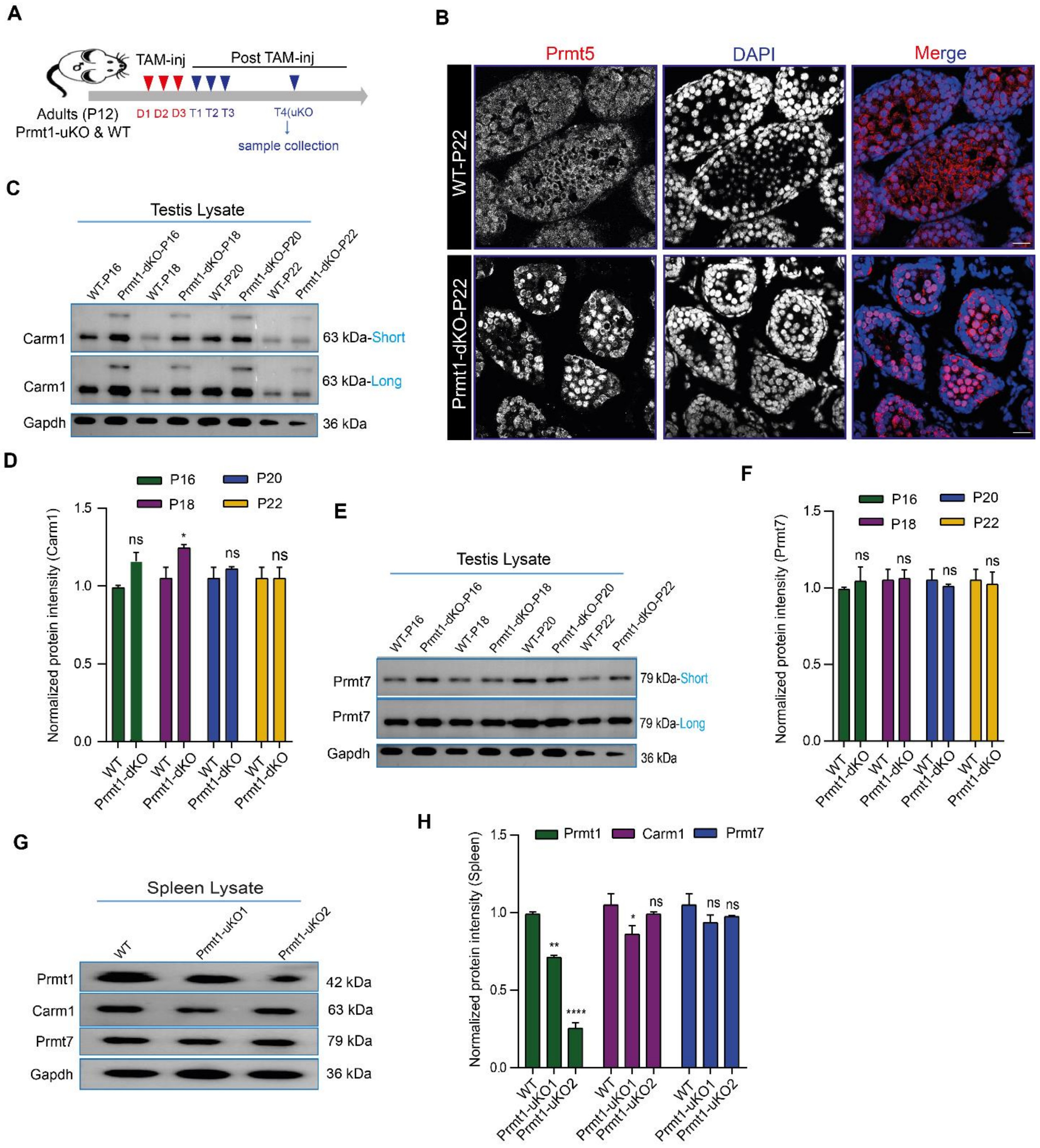
A
TAM-inj
Post TAM-inj
D1 D2 D3 T1 T2 T3
T4(uKO
sample collection
Adults (P12)
Prmt1-uKO & WT
B
Prmt5
DAPI
Merge
WT-P22
Prmt1-dKO-P22
C
Testis Lysate
WT-P16
Prmt1-dKO-P16
WT-P18
Prmt1-dKO-P18
WT-P20
Prmt1-dKO-P20
WT-P22
Prmt1-dKO-P22
Carm1
63 kDa-Short
Carm1
63 kDa-Long
Gapdh
36 kDa
D
P16
P18
P20
P22
Normalized protein intensity (Carm1)
WT
Prmt1-dKO
E
Testis Lysate
Prmt7
79 kDa-Short
Prmt7
79 kDa-Long
Gapdh
36 kDa
F
Normalized protein intensity (Prmt7)
G
Spleen Lysate
WT
Prmt1-uKO1
Prmt1-uKO2
Prmt1
42 kDa
Carm1
63 kDa
Prmt7
79 kDa
Gapdh
36 kDa
H
Prmt1
Carm1
Prmt7
Normalized protein intensity (Spleen)

Supplementary Fig. S3. Prmt1 depletion evokes an intrinsic compensatory expression of Prmt2 and Prmt6 responsible for the enhanced substrate ADMA methylation in testis

(A) Tamoxifen injection scheme in the P12 mice and tissue collection from Prmt1-uKO mice. D, Day. T, post-tamoxifen injection. (B) Immunostaining of H4R3me2s in testis sections from WT and Prmt1-dKO mice at P22. Scale bars: 20 μm. (C) Immunoblotting of Carm1 in WT and Prmt1-dKO mice at indicated time 'Figure S2C'. Gapdh served as a loading control. (D) Quantification of normalized protein band intensities for (b) (n = 2). *p<0.05. (E) Immunoblotting of Prmt7 in WT and Prmt1-dKO mice at indicated time 'Figure S2C'. (F) Quantification of normalized protein band intensities for 'D' (n = 2). (G) Immunoblotting of Prmt1, Carm1 and Prmt7 in WT and Prmt1-uKO mice at indicated time (Figure S2A). (H) Quantification of normalized protein band intensities for Prmt1, Carm1 and Prmt7 from WT and Prmt1-dKO mice (n = 2). **p<0.01, and ****p<0.0001.

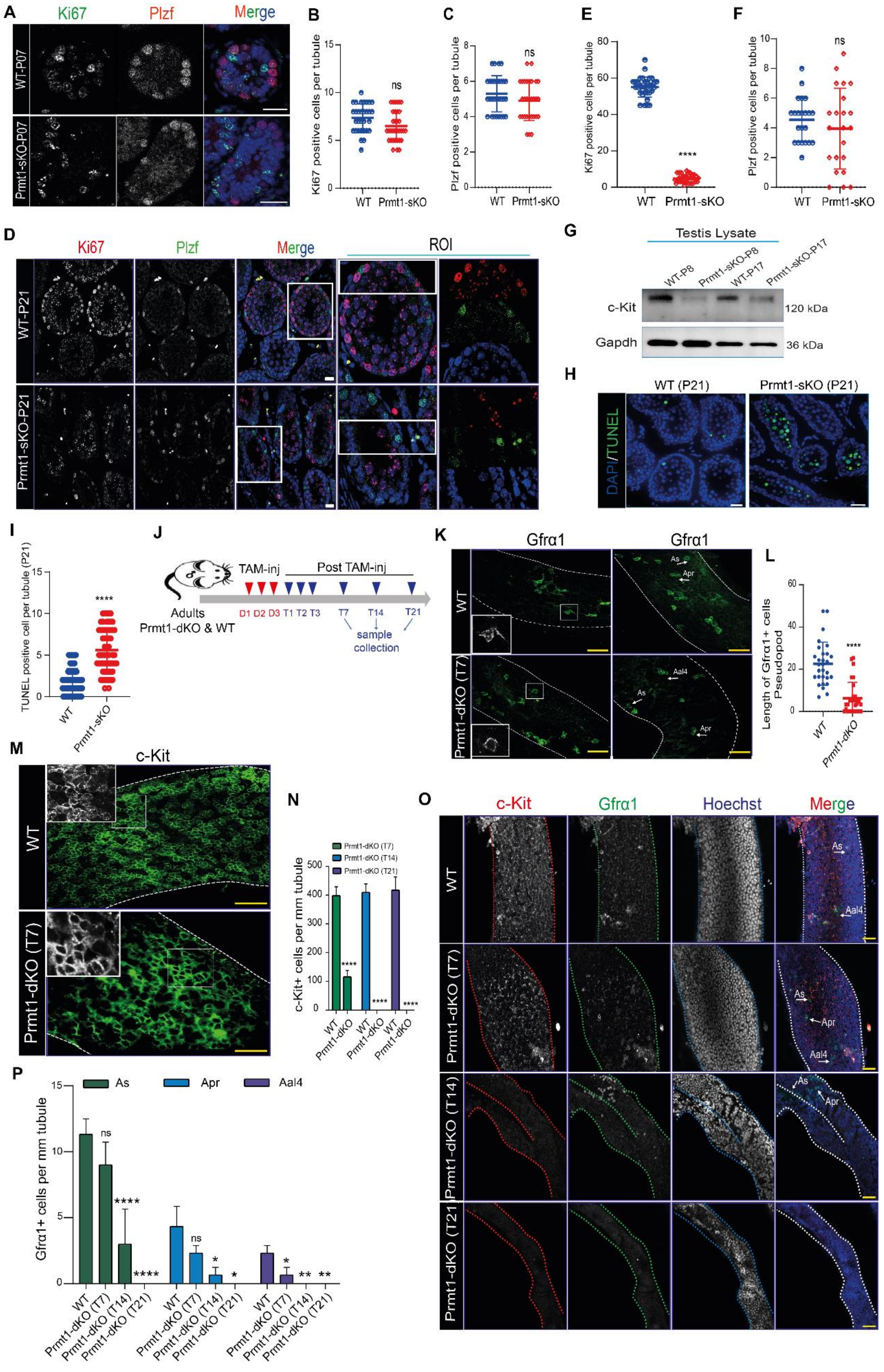

A
Ki67
Plzf
Merge
WT-P07
Prmt1-sKO-P07
B
Ki67 positive cells per tubule
WT
Prmt1-sKO
ns
C
Plzf positive cells per tubule
E
Ki67 positive cells per tubule
****
F
Plzf positive cells per tubule
D
ROI
WT-P21
Prmt1-sKO-P21
G
Testis Lysate
WT-P8
Prmt1-sKO-P8
WT-P17
Prmt1-sKO-P17
c-Kit
120 kDa
Gapdh
36 kDa
H
WT (P21)
Prmt1-sKO (P21)
DAPI/TUNEL
I
TUNEL positive cell per tubule (P21)
J
TAM-inj
Post TAM-inj
Adults
Prmt1-dKO & WT
D1 D2 D3 T1 T2 T3
T7
T14
T21
sample collection
K
Gfrα1
Prmt1-dKO (T7)
As
Apr
Aal4
L
Length of Gfrα1+ cells Pseudopod
Prmt1-dKO
M
c-Kit
N
Prmt1-dKO (T7)
Prmt1-dKO (T14)
Prmt1-dKO (T21)
c-Kit+ cells per mm tubule
O
Gfrα1
Hoechst
Prmt1-dKO (T14)
Prmt1-dKO (T21)
P
As
Apr
Aal4
Gfrα1+ cells per mm tubule
*
**

Supplementary Fig. S4. Prmt1 is required to maintain the developmental homeostasis of spermatogonial population through the first wave of spermatogenesis and in adult mouse testes

(A) Immunostaining by Ki67 and Plzf in testis sections from WT and Prmt1-sKO mice at P7. Scale bars, 20 μm. (B-C) Quantification of the numbers for Ki67- and Plzf-positive germ cells at P7, respectively (n=3). (D) Immunostaining by Ki67 and Plzf in testis sections from WT and Prmt1-sKO mice at P21. Scale bars, 20 μm. (E-F) Quantification of the numbers for Ki67- and Plzf-positive germ cells at P21, respectively (n=3). (G) Immunoblotting by c-Kit in testes from WT and Prmt1-sKO mice at P8 and P17. Gapdh served as a loading control. (H) TUNEL analysis of WT and Prmt1-sKO testes at P21. Scale bar, 20 μm. (I) Quantification of the numbers for TUNEL-positive cells. ****p<0.0001, mean±SEM, and n=3. (J) The scheme for tamoxifen injection and tissue collection in 'K,M and O'. (K) Pseudopod elongation by whole-mount staining of testicular tubules from WT and Prmt1-dKO mice at indicated times 'J'. As-single spermatogonia, Apr-paired spermatogonia, and Aal4-aligned-4 spermatogonia. (L) Pseudopod length quantification (n=3). ****p< 0.0001. (M) Whole-mount staining by c-Kit of testicular tubules from WT and Prmt1-dKO mice at the indicated time 'J'. (N) Quantification of the numbers for c-Kit positive cells at indicated times (n=3). ****p< 0.0001. (O) Whole-mount staining of testicular tubules by c-Kit and Gfrα1 in WT and Prmt1-dKO mice at the indicated times 'J'. (P) Quantification of the numbers for Gfrα1 (n=3). *p< 0.05, **p<0.01, ***p<0.001 and ****p< 0.0001.

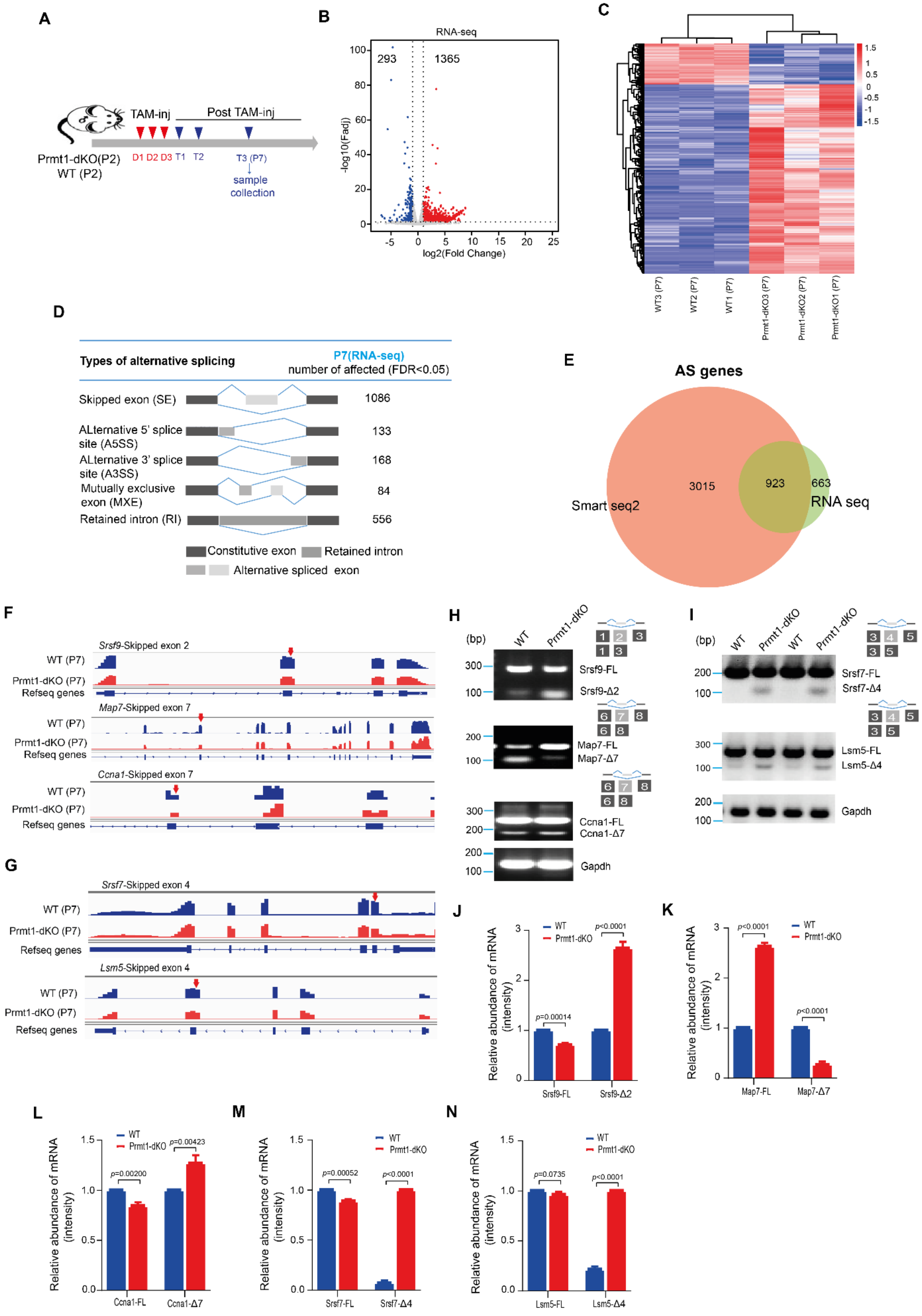

A
TAM-inj
Post TAM-inj
Prmt1-dKO(P2)
WT (P2)
D1 D2 D3 T1 T2
T3 (P7)
sample collection
B
RNA-seq
293
1365
-log10(Fadj)
log2(Fold Change)
C
WT3 (P7)
WT2 (P7)
WT1 (P7)
Prmt1-dKO3 (P7)
Prmt1-dKO2 (P7)
Prmt1-dKO1 (P7)
D
Types of alternative splicing
P7(RNA-seq)
number of affected (FDR<0.05)
Skipped exon (SE) 1086
ALternative 5' splice site (A5SS) 133
ALternative 3' splice site (A3SS) 168
Mutually exclusive exon (MXE) 84
Retained intron (RI) 556
Constitutive exon
Retained intron
Alternative spliced exon
E
AS genes
Smart seq2
3015
923
663
RNA seq
F
Srsf9-Skipped exon 2
Map7-Skipped exon 7
Ccna1-Skipped exon 7
WT (P7)
Prmt1-dKO (P7)
Refseq genes
G
Srsf7-Skipped exon 4
Lsm5-Skipped exon 4
H
(bp)
WT
Prmt1-dKO
Srsf9-FL
Srsf9-Δ2
Map7-FL
Map7-Δ7
Ccna1-FL
Ccna1-Δ7
Gapdh
I
Srsf7-FL
Srsf7-Δ4
Lsm5-FL
Lsm5-Δ4
Gapdh
J
Relative abundance of mRNA (intensity)
p=0.00014
p<0.0001
Srsf9-FL
Srsf9-Δ2
K
p<0.0001
p<0.0001
Map7-FL
Map7-Δ7
L
p=0.00200
p=0.00423
Ccna1-FL
Ccna1-Δ7
M
p=0.00052
p<0.0001
Srsf7-FL
Srsf7-Δ4
N
p=0.0735
p<0.0001
Lsm5-FL
Lsm5-Δ4

Supplementary Fig. S5. Prmt1 establishes the transcriptomic identity in spermatogonia

(A) The scheme for tamoxifen injection and tissue collection in ‘B’. (B) Volcano plot illustrating the dys-regulated genes in the Prmt1-dKO testis using bulk RNA-seq approach. (C) A heatmap illustrating the dys-regulated genes from WT and Prmt1-dKO testis using bulk RNA-seq. Fold change>2.0, and $p<0.05$ (Upregulated in red; Down-regulated in blue). (D) Statistical calculation of alternative splicing events identified by bulk RNA-seq. (E) Venn diagram presenting overlapped genes identified with aberrant splicing in the Prmt1-dKO testis between RNA-seq and Smart-seq2. (F) A snapshot of IGV browser showing mRNA expression levels for Srsf9, Map7, and Ccna1 in spermatogonia from WT and Prmt1-dKO mice; The red arrow indicates the splicing site. (G) A snapshot of IGV browser showing Srsf7 and Lsm5 mRNA expression. The red arrow indicates the splicing site. (H) RT-PCR verification for Srsf9, Map7, and Ccna1 genes showing AS pattern of exon skipping (Exon 2, 7, and 7), respectively in Prmt1-dKO testes; Gapdh was used as an internal control. (I) RT-PCR verification for Srsf7 and Lsm5 genes showing AS pattern of exon skipping (Exon 4) in Prmt1-dKO testes; Gapdh was used as an internal control. (J-N) Quantification of the expression for transcripts with skipped exon from ‘H and I’. FL, full length isoform, Δ represents skipped exon; (n=3). Two-tailed Student's t-test, the data are displayed as the mean±SEM.

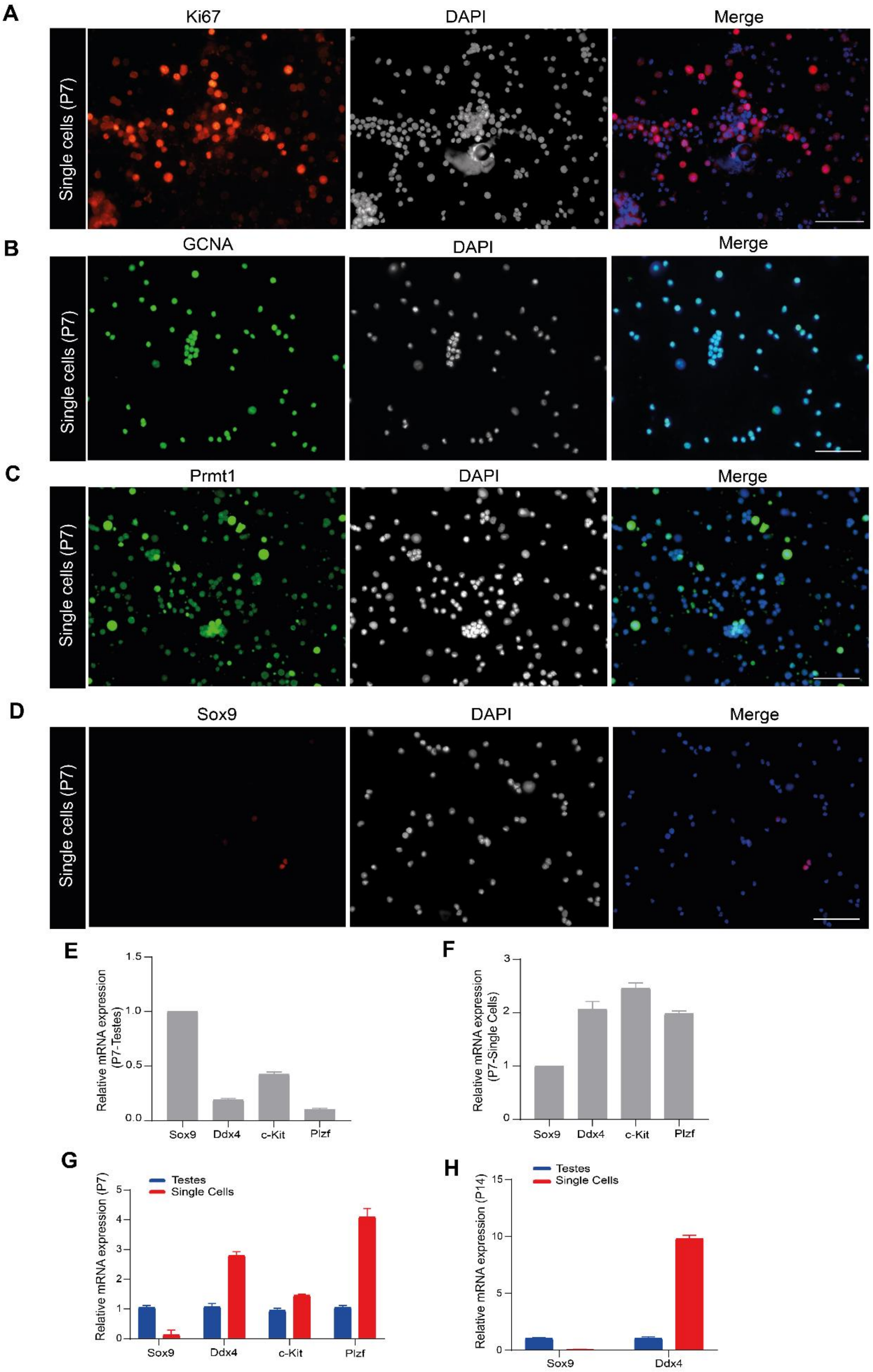
A
Ki67
DAPI
Merge
Single cells (P7)
B
GCNA
DAPI
Merge
Single cells (P7)
C
Prmt1
DAPI
Merge
Single cells (P7)
D
Sox9
DAPI
Merge
Single cells (P7)
E
Relative mRNA expression (P7-Testes)
1.5
1.0
0.5
0.0
Sox9
Ddx4
c-Kit
Plzf
F
Relative mRNA expression (P7-Single Cells)
3
2
1
0
Sox9
Ddx4
c-Kit
Plzf
G
Testes
Single Cells
Relative mRNA expression (P7)
5
4
3
2
1
0
Sox9
Ddx4
c-Kit
Plzf
H
Testes
Single Cells
Relative mRNA expression (P14)
15
10
5
0
Sox9
Ddx4

Supplementary Fig. S6. Ratios for germ cells and Sertoli cells in seminiferous epithelia-derived single-cell suspension at P7 and P14

(A-D) Immunostaining of Ki67, GCNA, Prmt1, and Sox9, respectively, in seminiferous epithelia-derived single cells from WT mice at P7. Scale bar, 50 µm. (E) Relative quantification of Sox9, Ddx4, c-Kit, and Plzf mRNA expression levels in testes (n = 2 mice per genotype). (F) Relative quantification of Sox9, Ddx4, c-Kit, and Plzf mRNA expression levels in single cells (n = 2 mice per genotype). (G) Relative quantification of Sox9, Ddx4, c-Kit, and Plzf mRNA expression levels in testes (P7) and single cells (P7) (n = 2 mice per genotype). (H) Relative quantification of Sox9 and Ddx4 mRNA expression levels in testes (P14) and single cells (P14) (n = 2 mice per genotype).

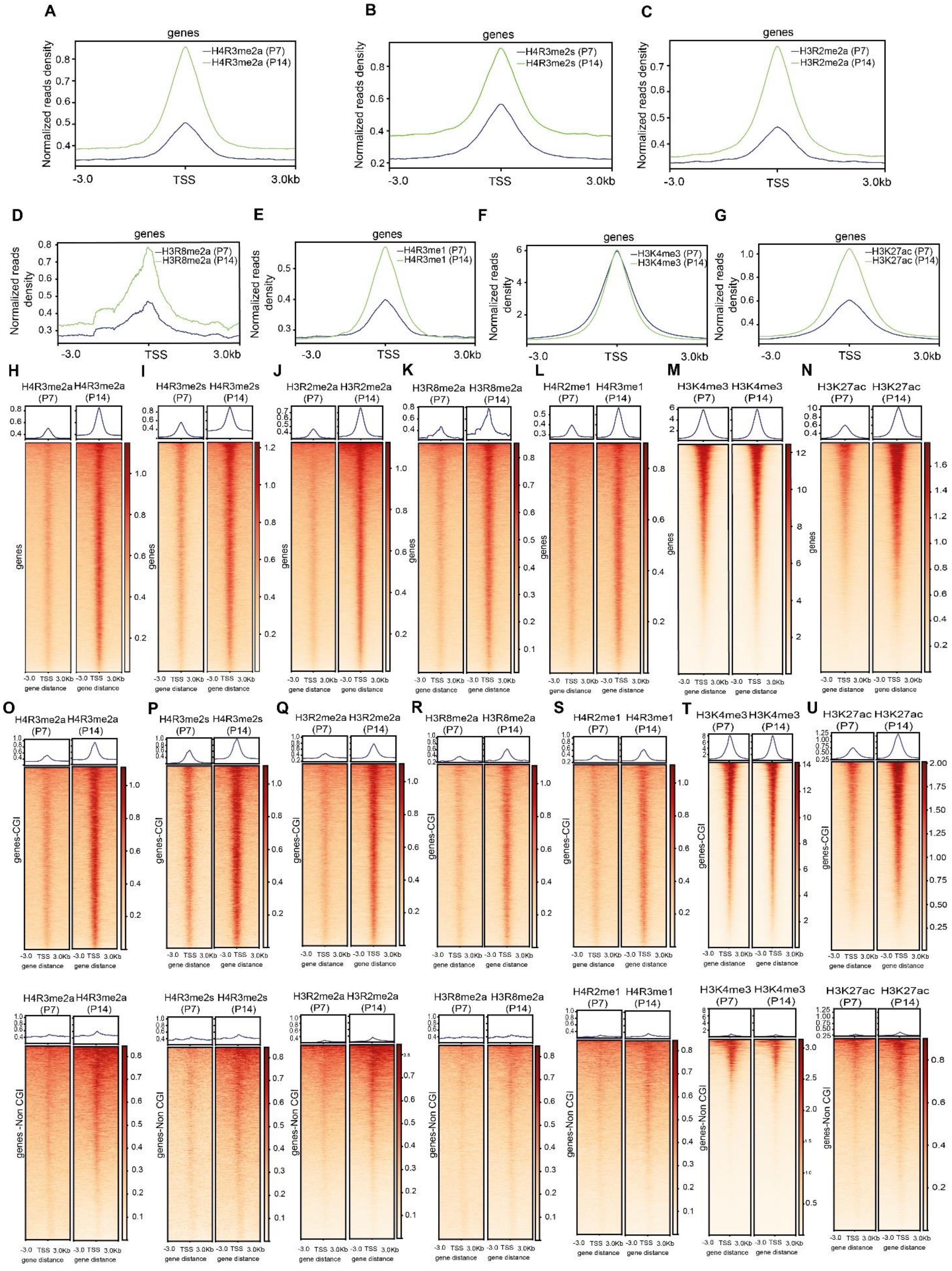
A
genes
Normalized reads density
H4R3me2a (P7)
H4R3me2a (P14)
-3.0
TSS
3.0kb
B
genes
H4R3me2s (P7)
H4R3me2s (P14)
C
genes
H3R2me2a (P7)
H3R2me2a (P14)
D
genes
H3R8me2a (P7)
H3R8me2a (P14)
E
genes
H4R3me1 (P7)
H4R3me1 (P14)
F
genes
H3K4me3 (P7)
H3K4me3 (P14)
G
genes
H3K27ac (P7)
H3K27ac (P14)
H
H4R3me2a (P7) H4R3me2a (P14)
I
H4R3me2s (P7) H4R3me2s (P14)
J
H3R2me2a (P7) H3R2me2a (P14)
K
H3R8me2a (P7) H3R8me2a (P14)
L
H4R2me1 (P7) H4R3me1 (P14)
M
H3K4me3 (P7) H3K4me3 (P14)
N
H3K27ac (P7) H3K27ac (P14)
genes
gene distance
O
P
Q
R
S
T
U
genes-CGI
genes-Non CGI

Supplementary Fig. S7. Global profiling for the genomic loci of histone arginine methyl marks in mouse testis at P7 and P14

(A-G) Tag density plots for histone mark enrichment at gene promoters for the five histone methylarginine marks (H4R3me2a, H4R3me2s, H4R3me1, H3R2me2a, and H3R8me2a), as well as H3K4me3 and H3K27ac, in mouse testis at P7 and P14. (H-N) Heatmap profile comparing the peak enrichment at gene promoters for the five histone methylarginine marks as well as H3K4me3 and H3K27ac in mouse testis at between P7 and P14. (O-U) Genes are categorized into two classes: non-CGI and CGI-containing genes. Top panel represents the peak enrichment for CGI-containing genes, while bottom panel indicates the peak enrichment for non-CGI genes.

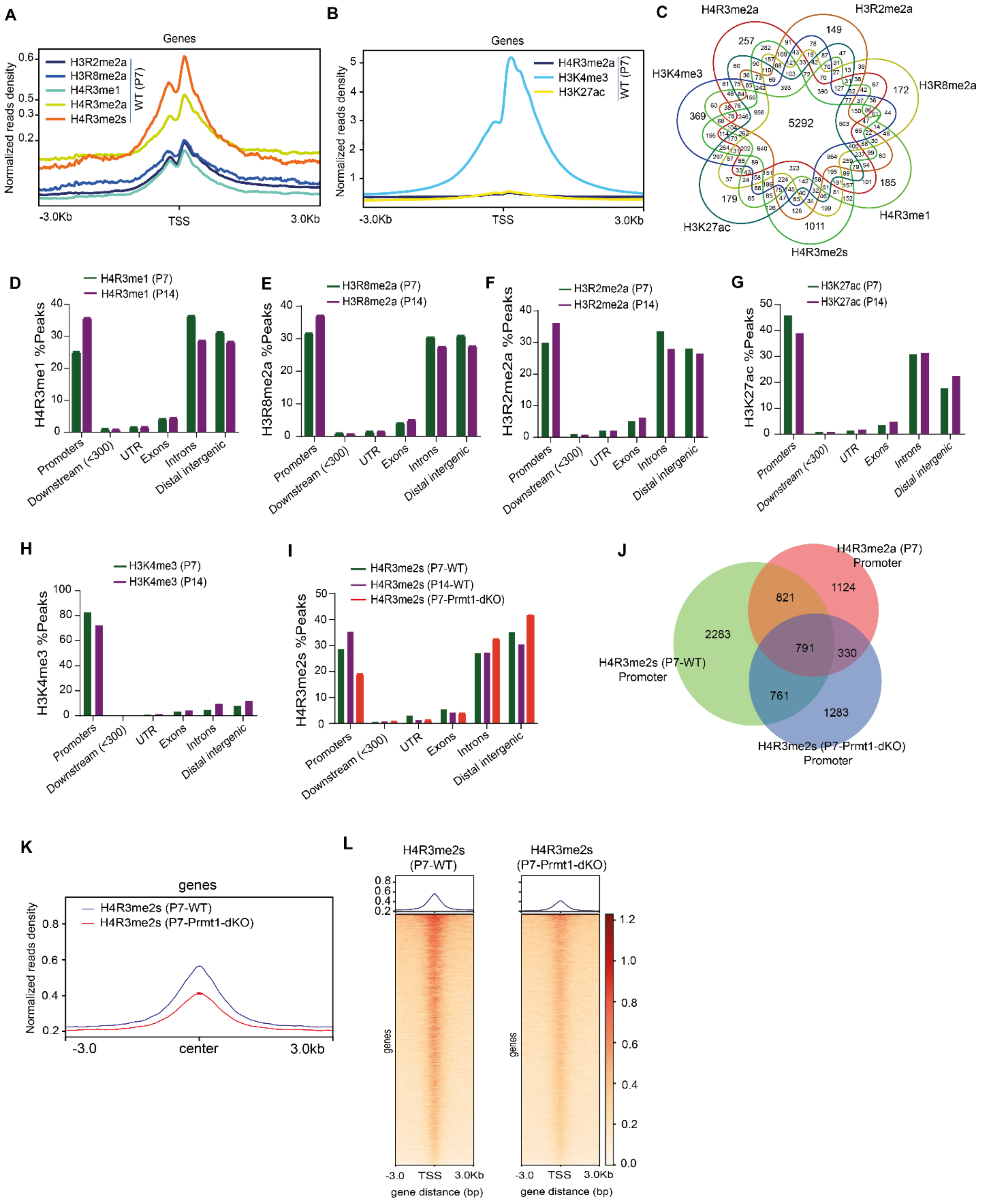

A
Genes
Normalized reads density
H3R2me2a
H3R8me2a
H4R3me1
H4R3me2a
H4R3me2s
WT (P7)
-3.0Kb
TSS
3.0Kb
B
Genes
Normalized reads density
H4R3me2a
H3K4me3
H3K27ac
WT (P7)
-3.0Kb
TSS
3.0Kb
C
H4R3me2a
H3R2me2a
H3K4me3
H3R8me2a
H4R3me1
H3K27ac
H4R3me2s
5292
D
H4R3me1 (P7)
H4R3me1 (P14)
H4R3me1 %Peaks
E
H3R8me2a (P7)
H3R8me2a (P14)
H3R8me2a %Peaks
F
H3R2me2a (P7)
H3R2me2a (P14)
H3R2me2a %Peaks
G
H3K27ac (P7)
H3K27ac (P14)
H3K27ac %Peaks
Promoters
Downstream (<300)
UTR
Exons
Introns
Distal intergenic
H
H3K4me3 (P7)
H3K4me3 (P14)
H3K4me3 %Peaks
I
H4R3me2s (P7-WT)
H4R3me2s (P14-WT)
H4R3me2s (P7-Prmt1-dKO)
H4R3me2s %Peaks
J
H4R3me2a (P7)
Promoter
H4R3me2s (P7-WT)
Promoter
H4R3me2s (P7-Prmt1-dKO)
Promoter
2283
821
1124
791
330
761
1283
K
genes
Normalized reads density
H4R3me2s (P7-WT)
H4R3me2s (P7-Prmt1-dKO)
-3.0
center
3.0kb
L
H4R3me2s
(P7-WT)
H4R3me2s
(P7-Prmt1-dKO)
genes
-3.0
TSS
3.0Kb
gene distance (bp)

Supplementary Fig. S8. Global profiling of histone arginine methyl marks by CUT&Tag profiling revealed the widespread genomic distribution in mouse testis at P7 and P14

(A)Tag density plots showing TSS enrichment for H4R3me2a, H4R3me2s, H4R3me1, H3R2me2a, and H3R8me2a in WT mouse testis at P7. (B) Tag density plots showing the comparison of TSS enrichment for H4R3me2a, H3K4me3, and H3K27ac in WT mouse testis at P7. (C) Overlapping of genes with peak enrichment identified for histone PTMs in WT mouse testis at P7. (D-H) Comparison of genomic enrichment distribution for H4R3me1, H3R8me2a, H3R2me2a, H3K27ac, and H3K4me3, respectively, in WT mouse testis at P7 and P14. (I) Comparison of genomic enrichment localization for H4R3me2s in testis of WT at P7, WT at P14, and Prmt1-dKO at P7. (J) Venn plot depicting the overlapping genes with enrichment at promoters in WT and Prmt1-dKO testis at P7 as indicated. (K) Averaged density plot for H4R3me2s enrichment at TSS in WT and Prmt1-dKO testis at P7. (L) Heat map profile of H4R3me2s showing the peak enrichment at TSS in WT and Prmt1-dKO testis at P7.

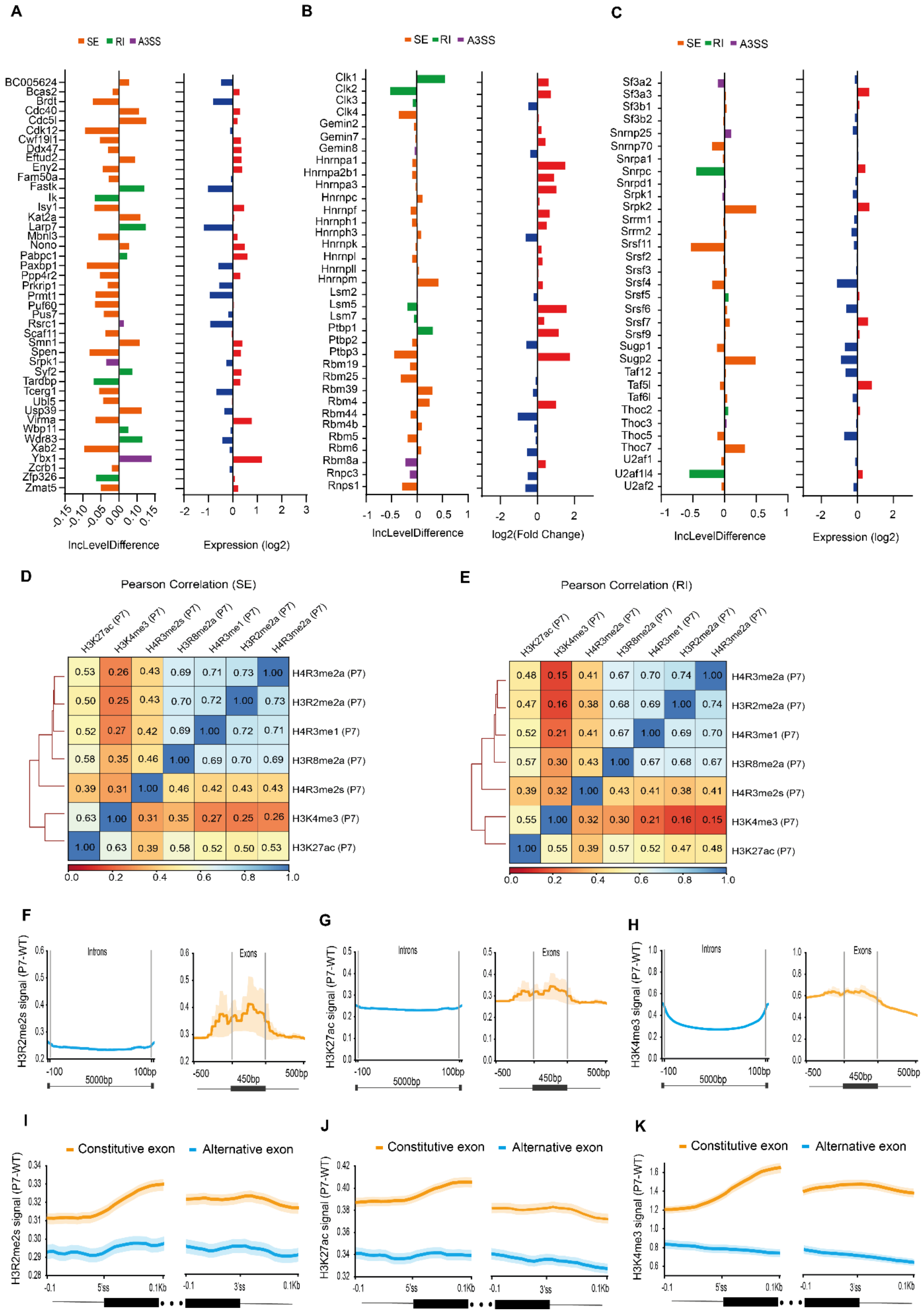
A
SE RI A3SS
BC005624 Bcas2 Brdt Cdc40 Cdc5l Cdk12 Cwf19l1 Ddx47 Eftud2 Eny2 Fam50a Fastk Ik Isy1 Kat2a Larp7 Mbnl3 Nono Pabpc1 Paxbp1 Ppp4r2 Prkrip1 Prmt1 Puf60 Pus7 Rsrc1 Scaf11 Smn1 Spen Srpk1 Syf2 Tardbp Tcerg1 Ubl5 Usp39 Virma Wbp11 Wdr83 Xab2 Ybx1 Zcrb1 Zfp326 Zmat5
IncLevelDifference
Expression (log2)
B
SE RI A3SS
Clk1 Clk2 Clk3 Clk4 Gemin2 Gemin7 Gemin8 Hnrnpa1 Hnrnpa2b1 Hnrnpa3 Hnrnpc Hnrnpf Hnrnph1 Hnrnph3 Hnrnpk Hnrnpl Hnrnpll Hnrnpm Lsm2 Lsm5 Lsm7 Ptbp1 Ptbp2 Ptbp3 Rbm19 Rbm25 Rbm39 Rbm4 Rbm44 Rbm4b Rbm5 Rbm6 Rbm8a Rnpc3 Rnps1
IncLevelDifference
log2(Fold Change)
C
SE RI A3SS
Sf3a2 Sf3a3 Sf3b1 Sf3b2 Snrnp25 Snrnp70 Snrpa1 Snrpc Snrpd1 Srpk1 Srpk2 Srrm1 Srrm2 Srsf11 Srsf2 Srsf3 Srsf4 Srsf5 Srsf6 Srsf7 Srsf9 Sugp1 Sugp2 Taf12 Taf5l Taf6l Thoc2 Thoc3 Thoc5 Thoc7 U2af1 U2af1l4 U2af2
IncLevelDifference
Expression (log2)
D
Pearson Correlation (SE)
H3K27ac (P7) H3K4me3 (P7) H4R3me2s (P7) H3R8me2a (P7) H4R3me1 (P7) H3R2me2a (P7) H4R3me2a (P7)
0.53 0.26 0.43 0.69 0.71 0.73 1.00 H4R3me2a (P7)
0.50 0.25 0.43 0.70 0.72 1.00 0.73 H3R2me2a (P7)
0.52 0.27 0.42 0.69 1.00 0.72 0.71 H4R3me1 (P7)
0.58 0.35 0.46 1.00 0.69 0.70 0.69 H3R8me2a (P7)
0.39 0.31 1.00 0.46 0.42 0.43 0.43 H4R3me2s (P7)
0.63 1.00 0.31 0.35 0.27 0.25 0.26 H3K4me3 (P7)
1.00 0.63 0.39 0.58 0.52 0.50 0.53 H3K27ac (P7)
E
Pearson Correlation (RI)
H3K27ac (P7) H3K4me3 (P7) H4R3me2s (P7) H3R8me2a (P7) H4R3me1 (P7) H3R2me2a (P7) H4R3me2a (P7)
0.48 0.15 0.41 0.67 0.70 0.74 1.00 H4R3me2a (P7)
0.47 0.16 0.38 0.68 0.69 1.00 0.74 H3R2me2a (P7)
0.52 0.21 0.41 0.67 1.00 0.69 0.70 H4R3me1 (P7)
0.57 0.30 0.43 1.00 0.67 0.68 0.67 H3R8me2a (P7)
0.39 0.32 1.00 0.43 0.41 0.38 0.41 H4R3me2s (P7)
0.55 1.00 0.32 0.30 0.21 0.16 0.15 H3K4me3 (P7)
1.00 0.55 0.39 0.57 0.52 0.47 0.48 H3K27ac (P7)
F
H3R2me2s signal (P7-WT)
Introns
Exons
-100 5000bp 100bp
-500 450bp 500bp
G
H3K27ac signal (P7-WT)
Introns
Exons
H
H3K4me3 signal (P7-WT)
Introns
Exons
I
Constitutive exon
Alternative exon
H3R2me2s signal (P7-WT)
5'ss
3'ss
J
Constitutive exon
Alternative exon
H3K27ac signal (P7-WT)
K
Constitutive exon
Alternative exon
H3K4me3 signal (P7-WT)

Supplementary Fig. S9. H4R3 methylation governs alternative splicing through directly regulating expression of splicing-related factors as well as indirect shaping of local chromatin signature

(A-C) Differential expression and inclusion level difference for RNA splicing-related genes. >0 indicates a difference in the degree of inclusion in Prmt1-dKO spermatogonia; <0 reflects inclusion level in WT. (D-E) A heatmap illustrating Pearson correlation of peak enrichment for histone modification marks in skipped exons (SE) and retained introns (RI) deduced from CUT&Tag profiling. (F-H) Averaged peak enrichment for H3R2me2a, H3K27ac and H3K4me3 from CUT&Tag signals for exons and introns. Average length for exons is 450 bp and for introns is 5 kb. (I-K) Enrichment comparison of the averaged peak intensity deduced from CUT&Tag for H3R2me2a, H3K27ac and H3K4me3, respectively, across the 100 bp flanking regions for alternative and constitutive exons.

Supplementary Table S1. Primer sequences for qPCR used in this study

Gene/Primer Name	Sequence	NCBI Reference Sequence
Etv5-F	CAAGTCCCTTTTATGGTCCCAG	NM_001358428.1
Etv5-R	ACTCTTCAGAATCGTGAGCCA	
Bmi1-F	AAATCCCCACTTAATGTGTGTCC	NM_001416912.1
Bmi1-R	CTTGCTGGTCTCCAAGTAACG	
Id4-F	ATGAACGACTGCTACAGTCG	NM_031166.3
Id4-R	GACTTTCTTGTTGGGCGGGAT	
Lhx1-F	TTCCTCTTGAACGTGTTGGAC	NM_008498.3
Lhx1-R	TCGGTCAGGTTGCATTTACATT	
Gfra1-F	GCACCAAGTACCGCACACT	NM_001410348.1
Gfra1-R	GCGGCAGTTGTAGAGAGACTTC	
Lin28a-F	GGCATCTGTAAGTGGTTCAACG	NM_145833.1
Lin28a-R	GCCAGTGACACGGATGGATT	
Nanos3-F	ATGGGGACTTTCAATCTTTGGA	NM_194059.2
Nanos3-R	GTTTGCAGAATGAACATAAGCGT	
Sox3-F	CCTTCTGAGTTGATACGGTTGTC	NM_173384.2
Sox3-R	GGTCGCTTCACATGACCATTTC	
Pou5f1-F	CACCATCTGTCGCTTCGAGG	NM_013633.3
Pou5f1-R	AGGGTCTCCGATTTGCATATCT	
Sohlh1-F	AGAGCGCGTTGTCATTCAGT	NM_001001714.2
Sohlh1-R	CATGCCGGAAGATTCAGGG	
Rarγ-F	AAGTACACCACGAACTCCAGT	NM_011244.5
Rarγ-R	TTCGCAAACTCCACAATCTTGA	
Plzf-F	ACTCTGCGGAAAACGGTTC	NM_001033324.3
Plzf-R	GTGCCAGTATGGGTCTGTCT	
Sall4-F	CCCTGGGAACTGCGATGAAG	NM_175303.4
Sall4-R	TCAGAGAGACTAAAGAACTCGGC	
Dmrtb1-F	AATCCTGAGAGAGAGCCGTTC	NM_019872.2
Dmrtb1-R	GCCGGAAGCCTCTTTGTTGA	
Dnmt3b-F	CGTTAATGGGAACTTCAGTGACC	NM_001003961.5
Dnmt3b-R	CTGCGTGTAATTCAGAAGGCT	
Stra8-F	ACAACCTAAGGAAGGCAGTTTAC	NM_009292.3
Stra8-R	GACCTCCTCTAAGCTGTTGGG	
c-Kit-F	GGCCTCACGAGTTCTATTTACG	NM_001122733.1
c-Kit-R	GGGGAGAGATTTCCCATCACAC	
Ccnd1-F	GCGTACCCTGACACCAATCTC	NM_001379248.1
Ccnd1-R	ACTTGAAGTAAGATACGGAGGGC	

Prdm9-F	AGATACAGGGAAATTCGAGTGGA	NM_144809.3
Prdm9-R	GGTAACACATGAAAGCTGGTCT	
Brca1-F	CCACAAAGTGTGACCACATA	NM_009764.3
Brca1-R	CCTCATTCAAACGCTCACAAG	
Clk4-F	GTTCCAAGACATTACCATAGA	NM_007714.6
Clk4-R	TCACCTAAAGTGTCCACGATT	
Cenpt-F	GCTCTTGAGATAGTAGAGAAG	NM_177150.2
Cenpt-R	CAGCAGCAATAAGTCCTCTG	
Ccna1-F	CATCAGGACTGAGAATTTGGC	NM_001305221.1
Ccna1-R	CTCCCTGATTGCTTGCTGCG	
Srsf7-F	ATAGGCCACCTGCCCGTCGT	NM_001404106.1
Srsf7-R	ATCGGCGAGGAGATGCTGAT	
Srsf9-F	GAGAAGGACCTCGAGGACTTGT	NM_025573.3
Srsf9-R	GCTTCTCGCATGTGATCTTTCAG	
Lsm5-F	TAACGCGACCACGAACCCGTC	NM_025520.3
Lsm5-R	TCATACTTCAGGCCCTTCTCCT	
Map7-F	TGCTAAACTCTCCAGATAGAG	NM_001198635.1
Map7-R	TGCTGAACGAGGACAAATGG	
Prmt1-F	TACTACTTTGACTCCTATGCCCA	NM_019830.3
Prmt1-R	ATGCCGATTGTGAAACATGGA	
Prmt2-F	AGCGCCGAGAAAGACTACC	NM_133182.3
Prmt2-R	GGCCTTGAAAAGAACTCCTTGA	
Prmt3-F	GGGATCGGTCTACCCTGACAT	NM_133740.2
Prmt3-R	AGCTTCCGGTATAACTGCTTTC	
Carm1-F	TGGAATGCCTACTGCCTACG	NM_021531.6
Carm1-R	CATCCGGGAGTGGGTGTGAT	
Prmt5-F	GCCAAGCAGGGGTTTGATTTCCT	NM_013768.3
Prmt5-R	AGCTTTCCCACAATTAGCGTATTCC	
Prmt6-F	GGTGCCGGTGGAACAAGATA	NM_178891.5
Prmt6-R	CTCCCACTTTGTAGCGCAGA	
Prmt7-F	AGTGGATCGCTTCCCACAGC	NM_145404.1
Prmt7-R	TCCCGTGGTAGGATTGGCAC	
Prmt8-F	GCATGAAACACTCCTCCCGC	NM_201371.2
Prmt8-R	CACCTCGGTGCTTTCGACT	
Prmt9-F	CTCGCCTGTGATGTAGTGGC	NM_001081240.3
Prmt9-R	TACACCTGCATCGACAGTTTCTG	
Gapdh-F	AGGTCGGTGTGAACGGATTTG	NM_001289726.2
Gapdh-R	TGTAGACCATGTAGTTGAGGTCA	

References

1. Ernst, C., Eling, N., Martinez-Jimenez, C.P., Marioni, J.C. and Odom, D.T. (2019) Staged developmental mapping and X chromosome transcriptional dynamics during mouse spermatogenesis. *Nat Commun*, **10**, 1251.
